# Lysophosphatidic acid mediates skeletal muscle fibrosis in denervation via activation of YAP/TAZ

**DOI:** 10.1172/jci.insight.198388

**Published:** 2026-04-22

**Authors:** Meilyn Cruz-Soca, Adriana Córdova-Casanova, Jennifer Faundez-Contreras, Nicolás W. Martínez, Francesca Vaccaro-Rivera, Sebastián Bazaes-Astorga, Cristian Gutiérrez-Rojas, Felipe S. Gallardo, Daniela L. Rebolledo, Felipe A. Court, Jerold Chun, Carlos P. Vio, Soledad Matus, Juan Carlos Casar, Enrique Brandan

**Affiliations:** 1 Facultad de Medicina, Universidad San Sebastián, Providencia, Chile.; 2 Centro Científico y Tecnológico de Excelencia Ciencia & Vida, Huechuraba, Chile.; 3 Escuela de Kinesiología, Facultad de Ciencias, Pontificia Universidad Católica de Valparaíso, Valparaíso, Chile.; 4 Center for Aging Research and Healthy Longevity, Faculty of Sciences, Universidad Mayor, Huechuraba, Chile.; 5 Buck Institute for Research on Aging, Novato, California, USA.; 6 Sanford Burnham Prebys Medical Discovery Institute, La Jolla, California, USA.; 7 Facultad de Ciencias, Universidad San Sebastián, Providencia, Chile.; 8 Departmento de Neurología, Facultad de Medicina, Pontificia Universidad Católica de Chile, Santiago, Chile.

**Keywords:** Cell biology, Muscle biology, Fibrosis, Signal transduction, Skeletal muscle

## Abstract

Lysophosphatidic acid (LPA) is a bioactive lipid that signals through G protein–coupled receptors (LPA_1–6_) and regulates multiple cellular processes, including fibrosis. Although LPA signaling has been implicated in fibrotic diseases in several organs, its role in skeletal muscle remains unclear. Here, we show that LPA/LPA_1_ signaling promotes fibrogenesis after sciatic nerve transection. Denervation induces differential expression of LPA signaling axis components and a transient early increase in intramuscular LPA levels. Pharmacological inhibition of LPA_1/3_ with Ki16425, or genetic deletion of LPA_1_, reduces extracellular matrix accumulation and expansion of fibro/adipogenic progenitors (FAPs) in denervated muscle. Although LPA blockade suppresses atrophy-related gene expression, it does not fully preserve myofiber size. Mechanistically, denervation increases YAP/TAZ expression, nuclear localization in FAPs, and transcriptional activity, effects that are attenuated by LPA axis inhibition. Furthermore, pharmacological inhibition of YAP/TAZ with verteporfin reduces fibrosis after denervation, supporting their role as critical downstream mediators. Finally, transient denervation activates the LPA axis, promotes muscle fibrosis, reduces axonal density in the sciatic nerve, and increases neuromuscular junction instability, effects reversed by Ki16425. Together, these findings identify the LPA/LPA_1_/YAP/TAZ pathway as a key driver of denervation-induced muscle fibrosis and a potential therapeutic target in neuromuscular disorders.

## Abstract 2

Blocking LPA–LPA1–YAP/TAZ signaling reduces muscle fibrosis and nerve damage after injury, revealing a potential treatment strategy for neuromuscular disorders.

## Introduction

Skeletal muscle denervation is a pathological condition characterized by the loss of neural input, with the consequent interruption of synaptic transmission from motor neurons. It can result from traumatic injuries, such as peripheral nerve compression or transection, or as a feature in pathological conditions, including neuromuscular disorders like amyotrophic lateral sclerosis (ALS) and muscular dystrophies (1–5), common metabolic diseases such as diabetes, and even aging (5–7). Transection of the sciatic nerve is widely used as an experimental model to study the effects of the loss of neuromuscular transmission under controlled conditions (3, 8–11). After denervation, dependent hindlimb muscles undergo profound alterations, including atrophy, mitochondrial dysfunction, and increased autophagy (9, 11).

Loss of innervation also initiates a series of cellular and molecular responses that ultimately lead to excessive extracellular matrix (ECM) accumulation, resulting in fibrosis (12). This fibrotic process involves the upregulation of profibrotic mediators, such as TGF-β and connective tissue growth factor (CTGF/CCN2), together with the activation, proliferation, and expansion of fibro/adipogenic progenitors (FAPs) (8, 13, 14). These cells differentiate into myofibroblasts, which are responsible for excessive ECM deposition and increased tissue stiffness (15, 16). Fibrotic mechanisms are also prominently activated in chronic neuromuscular disorders, including in patients with Duchenne muscular dystrophy (DMD) (17), in the *mdx* murine model of DMD (13, 14), and in symptomatic ALS transgenic mice (hSOD1^G93A^) (4, 18).

Fibrosis is closely related to other pathological features in skeletal muscle, including degeneration, inflammation, and atrophy, ultimately exacerbating disease progression (19, 20). Given its detrimental effects, identifying novel molecular mediators involved in the onset and maintenance of muscle fibrosis is a critical step toward developing therapeutic strategies, particularly in neuromuscular disorders, where reducing fibrosis has been shown to improve muscle contractile function (4, 21). Inhibiting fibrosis, a palliative approach to disease, may also improve the efficacy of treatments with curative intention, such as cell and gene therapies (22, 23). For instance, studies in liver fibrosis have demonstrated that its mitigation improves the efficiency of gene transfer to hepatocytes, leading to better gene delivery outcomes (24, 25). Similarly, in skeletal muscle, reducing ECM deposition restores microcirculation and enables efficient cell therapy in aged dystrophic mice (23).

Lysophosphatidic acid (LPA) has emerged as a key signaling lipid among the various molecular mediators involved in fibrosis. LPA is a bioactive phospholipid composed of a phosphate, glycerol, and a fatty acid chain (26). It signals through 6 G protein–coupled receptors (LPARs), designated as LPA_1_ to LPA_6_, which regulate diverse cellular responses, including proliferation, survival, migration, and differentiation (27–30). LPA is mainly synthesized extracellularly by autotaxin (ATX), a secreted lysophospholipase that hydrolyzes lysophosphatidylcholine to produce equimolar amounts of LPA and choline (31). LPA is degraded by 3 membrane-bound lipid phosphate phosphatases, which regulate its bioavailability (32). Dysregulated LPA signaling has been implicated in multiple pathological conditions, such as cancer, chronic inflammation, and fibrosis (33, 34). In fibrotic diseases, LPA promotes fibroblast activation and differentiation into myofibroblasts (30, 35). Consistently, pharmacological blockade of LPA signaling has demonstrated antifibrotic effects in animal models of skin, liver, kidney, and pulmonary fibrosis (35–40).

LPA contributes to increased muscle atrophy, fibrosis, and fatty infiltration after rotator cuff tears (41). Components of the LPA axis are present in skeletal muscle, with LPA_1_ and LPA_6_ being the most abundantly expressed receptors. Intramuscular injection of exogenous LPA recapitulates several features of skeletal muscle fibrosis, primarily through LPA_1_, including increased ECM deposition and expansion of the FAP population (42). However, most available evidence linking LPA signaling to skeletal muscle fibrosis is based on exogenous LPA administration. Whether denervation alone is sufficient to activate endogenous LPA signaling in skeletal muscle, how components of the LPA axis are regulated after nerve injury, and which downstream signaling pathways contribute to the ensuing fibrotic response remain largely unexplored.

To further dissect the downstream signaling pathways mediating the fibrotic response in denervated skeletal muscle, attention was focused on the transcriptional coactivators Yes-associated protein 1 (YAP) and transcriptional coactivator with PDZ-binding motif (TAZ) (43). YAP/TAZ have been identified as downstream effectors of LPA signaling in multiple cellular contexts (44–46). Moreover, YAP/TAZ regulates fibrogenic responses in several organs, including the lung, kidney, heart, and liver (47–51). When phosphorylated, these proteins are retained in the cytoplasm (52) or targeted for degradation (53). In their active (non-phosphorylated) state, YAP and TAZ translocate to the nucleus, interacting with TEA domain family members (TEAD). YAP and TAZ act as transcriptional coactivators of genes involved in cell proliferation, apoptosis, cell fate determination (54), and fibrotic processes, including the induction of CCN2 (55). Our group has demonstrated that LPA-induced FAP activation and migration in vitro requires YAP/TAZ (56, 57). In addition, denervation leads to increased YAP expression and its nuclear accumulation in FAPs (58). Recently, Gallardo et al. showed that inhibiting YAP/TAZ signaling limits FAP expansion and reduces muscular fibrosis in a model of acute muscle injury (59).

In this study, we demonstrate the involvement of the LPA axis, mediated by LPA_1_, in a murine model of denervation-induced muscular fibrosis. Denervation leads to a coordinated deregulation of LPA axis components, including a marked increase in LPA_1_ expression and a transient early rise in intramuscular LPA levels. We found that genetic deletion and pharmacological inhibition of LPA_1_ significantly reduce fibrosis after denervation. Moreover, LPA is required for FAP accumulation and YAP/TAZ expression, transcriptional activity, and accumulation within FAP nuclei in denervated muscle. Additionally, transient denervation by crush is sufficient to activate the LPA axis, trigger a fibrotic response in skeletal muscle, reduce axonal density in the sciatic nerve, and increase denervation of neuromuscular junctions, effects that are reversed by treatment with Ki16425. Finally, we demonstrate that YAP/TAZ activity is necessary for the fibrotic response to denervation. Altogether, our results identify the LPA/LPA_1_/YAP/TAZ axis as a critical mediator of muscle fibrosis and peripheral nerve integrity, highlighting its potential as a therapeutic target in neuromuscular disorders.

## Results

### LPA signaling axis components are differentially expressed in denervated skeletal muscles.

**Figure 1:**
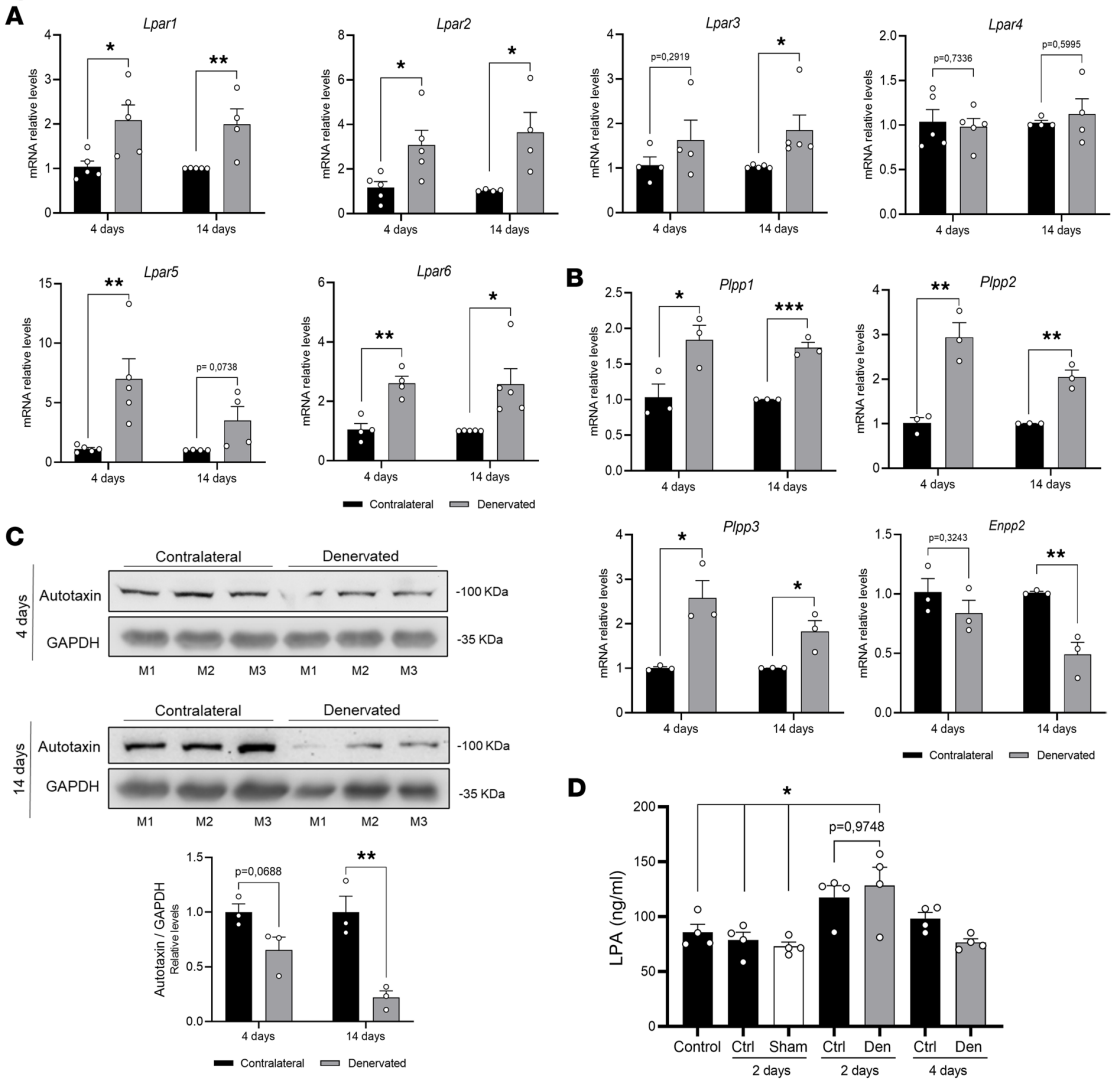
LPA signaling axis components are differentially expressed in denervated skeletal muscles. (**A** and **B**) Comparison of mRNA levels in GST muscles 4 days and 2 weeks after denervation against their contralateral controls. (**A**) RT-qPCR analysis of *Lpar1* to *Lpar6* expression (*n* = 4 or 5). (**B**) RT-qPCR analysis of lipid phosphate phosphatases 1, 2, and 3 (*Plpp1*, *Plpp2,* and *Plpp3*) and ATX (*Enpp2*) mRNA expression (*n* = 3). (**C**) Western blot analysis of ATX protein levels (*n* = 3). *P < 0.05, **P < 0.01, ***P < 0.001 with 2-tailed Student′s *t* test. Values are shown as mean ± SEM. (**D**) LPA levels in the GST muscle (*n* = 4), Ctrl (contralateral). *P < 0.05 with 1-way ANOVA test. Values are shown as mean ± SEM.

To investigate the involvement of the LPA signaling axis in skeletal muscle after denervation, we experimentally analyzed the expression of key components of this pathway in vivo. Specifically, we assessed mRNA levels of LPA receptors (*Lpar1–6*) and enzymes involved in LPA synthesis (ATX, *Enpp2*) and degradation (lipid phosphate phosphatases, *Plpp1–3*) in gastrocnemius (GST) muscles at 4 days and 2 weeks after sciatic nerve transection. We observed an upregulation of mRNA expression for 4 of the 6 LPARs in the denervated muscles as compared with their contralateral counterparts. The upregulation included 2 abundantly expressed receptors, *Lpar1* and *Lpar6*, and 2 receptors with lower expression levels, *Lpar2* and *Lpar3* (42) (Figure 1A). Denervation also led to increased mRNA expression of the lipid phosphate phosphatase enzymes (*Plpp1–3*) and downregulation of ATX (*Enpp2*) expression (Figure 1B). Consistently, ATX protein levels were reduced in denervated muscles (Figure 1C).

In addition to transcriptional changes in components of the LPA signaling axis, we directly quantified LPA levels in GST muscle at early time points after denervation. LPA concentrations were measured by ELISA in non-operated control mice, sham-operated mice at 2 days (contralateral and sham-operated GST muscles), and mice subjected to sciatic nerve transection for 2 or 4 days (contralateral and denervated GST muscles). LPA levels were significantly increased in denervated GST muscle at 2 days after denervation compared with both non-operated controls and sham-operated GST muscle (Figure 1D). By 4 days after denervation, LPA levels returned to values comparable to basal conditions. Although not reaching statistical significance, contralateral GST muscle from mice denervated for 2 days showed a trend toward increased LPA levels; this effect was not observed in GST muscle from sham-operated animals. Collectively, these results indicate that denervation is associated with an early and transient increase in intramuscular LPA levels in GST muscle.

### Pharmacological inhibition of LPA_1_ and LPA_3_ reduces the fibrotic response after denervation.

**Figure 2:**
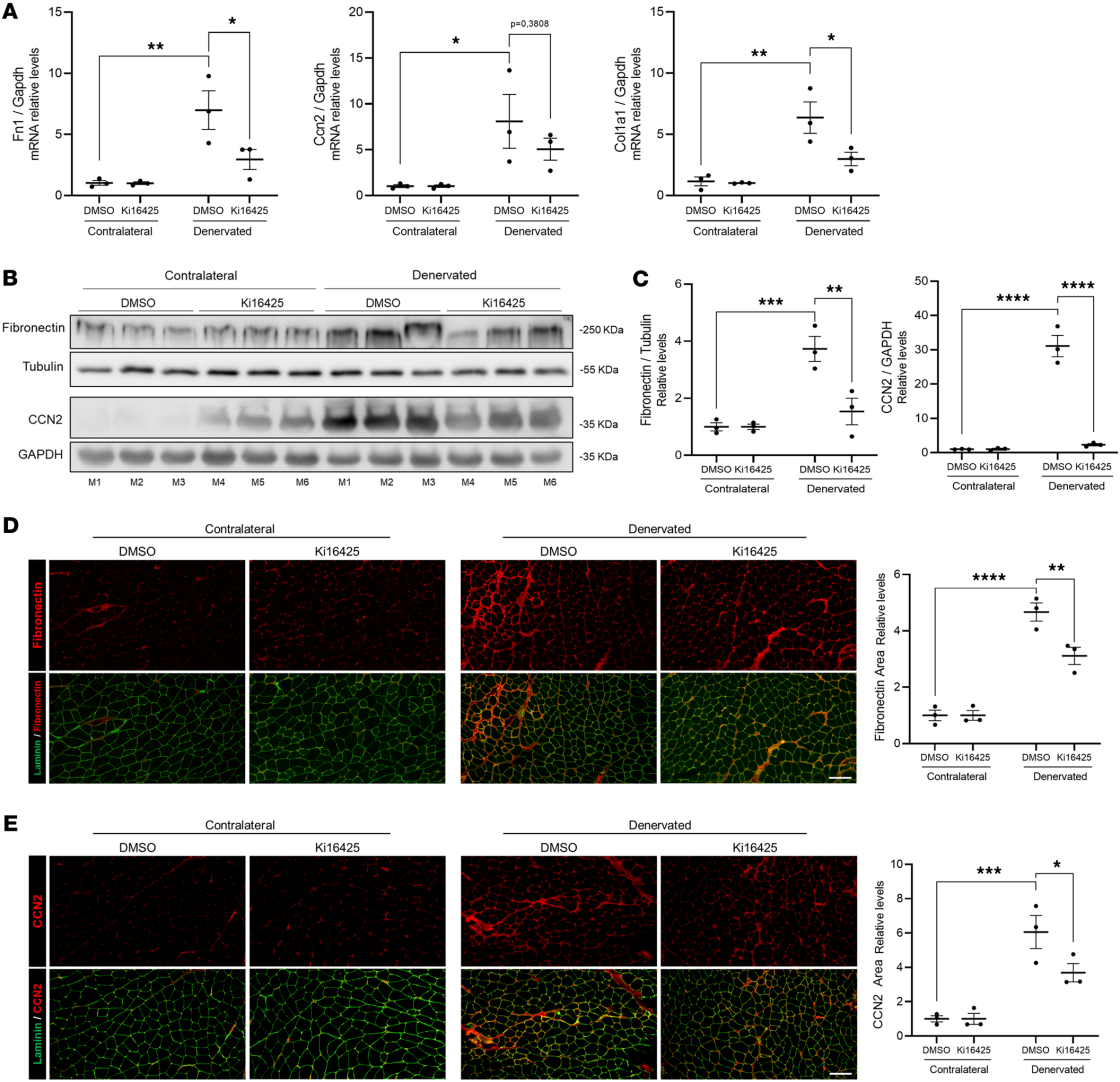
Pharmacological inhibition of LPA_1_ and LPA_3_ reduces the fibrotic response after denervation. For 3 days before unilateral sciatic denervation, 6-month-old C57Bl/6J mice were treated with vehicle (DMSO) (*n* = 3) or Ki16425 (*n* = 3). Skeletal muscles from both hindlimbs were collected 2 weeks after denervation. (**A**) Fibronectin (*Fn1*), CCN2 (*Ccn2)*, and collagen I (*Col1a1*) mRNA levels were measured by RT-qPCR. (**B**) GST homogenates were subjected to SDS-PAGE and immunoblotted for fibronectin and CCN2. Tubulin and GAPDH were used as loading controls. (**C**) Quantification of **B**. (**D**) Frozen tissue cross-sections from contralateral and denervated GST were subjected to immunofluorescence for the detection of fibronectin (red) and laminin (green). Scale bar: 100 μm. Quantification of fibronectin-positive area. (**E**) Representative immunofluorescence images showing CCN2 (red) and laminin (green). Scale bar: 100 μm. Quantification of CCN2-positive area (**E**). *P < 0.05, **P < 0.01, ***P < 0.001, ****P < 0.0001 with 1-way ANOVA test. Values are shown as mean ± SEM.

To evaluate whether LPA signaling is involved in denervation-induced fibrosis, we treated mice with the LPA_1_ and LPA_3_ receptor inhibitor Ki16425 (60). The inhibitor was administered intraperitoneally for 3 days before denervation and then daily for 2 weeks, after which fibrosis-related markers were evaluated (8). Our results showed that fibronectin (*Fn1*), CCN2 (*Ccn2*), and collagen I (*Col1a1*) mRNA levels increased in the denervated limb, and the inhibition of LPA_1_ and LPA_3_ with Ki16425 significantly reduced *Fn1* and *Col1a1* (Figure 2A). We also observed reduced accumulation of fibronectin and CCN2 after denervation by Western blot analysis of muscle extracts from mice treated with Ki16425, compared with those treated with DMSO (Figure 2, B and C). Given the established involvement of CCN family members in fibrotic processes (61, 62), we next assessed the expression of additional CCN genes. *Ccn3* expression was not altered by denervation or LPA_1_ inhibition, whereas *Ccn4* mRNA levels were significantly increased after denervation and reduced by Ki16425 treatment (Supplemental Figure 1, A and B; supplemental material available online with this article; https://doi.org/10.1172/jci.insight.198388DS1).

Decreased levels of fibronectin (Figure 2D) and CCN2 (Figure 2E) were observed by indirect immunofluorescence in cross-sections of denervated muscles from mice treated with Ki16425; reduced total collagen levels were also observed, as demonstrated by Sirius red staining (Supplemental Figure 2, A and B). Our results suggest that signaling through LPA_1_/LPA_3_ is required for the skeletal muscle fibrotic response induced by denervation.

### Lpar1 ablation prevents the skeletal muscle fibrotic response to denervation.

**Figure 3:**
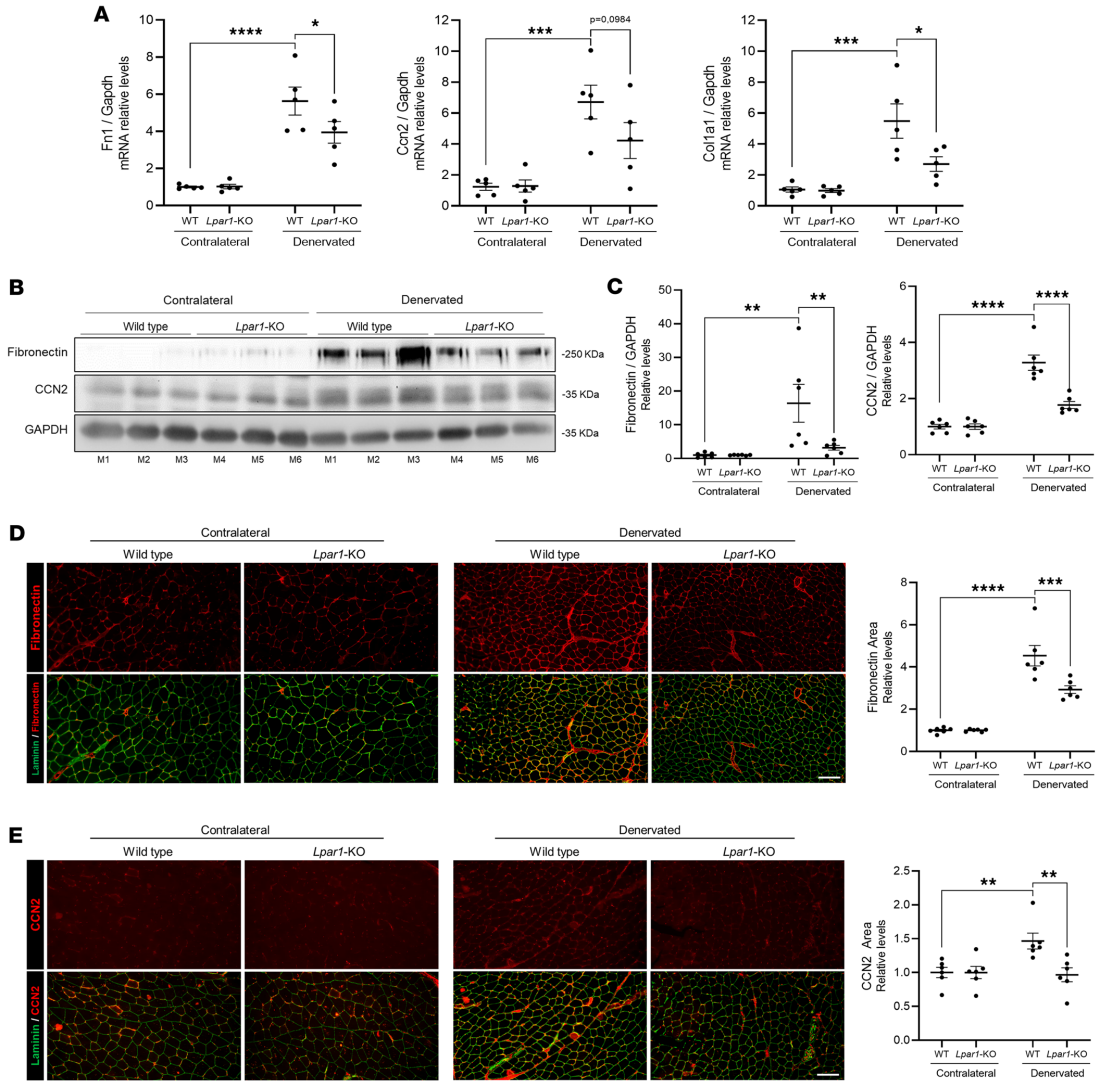
*Lpar1* ablation prevents the skeletal muscle fibrotic response to denervation. Six-month-old BALB/c (*n* = 5–6) and *Lpar1*-KO (*n* = 5–6) mice subjected to unilateral sciatic nerve transection. Skeletal muscles from both hindlimbs were collected 2 weeks after denervation. (**A**) Fibronectin (*Fn1*), CCN2 (*Ccn2)*, and collagen I (*Col1a1*) mRNA levels were measured by RT-qPCR. (**B**) Western blot analysis of fibronectin and CCN2. GAPDH was used as the loading control. (**C**) Quantification of **B**. (**D**) Immunofluorescence of fibronectin (red) and laminin (green). Scale bar: 100 μm. Quantification of fibronectin-positive area (**E**) Representative immunofluorescence images of CCN2 (red) and laminin (green). Scale bar: 100 μm. Quantification of CCN2-positive area. *P < 0.05, **P < 0.01, ***P < 0.001, ****P < 0.0001, with 1-way ANOVA test. Values are shown as mean ± SEM.

As a second strategy to investigate the role of LPA signaling in the fibrotic response induced by denervation, we used *Lpar1*-KO mice. This approach was selected based on several considerations: (a) LPA_1_ is the most studied LPAR in the development of renal fibrosis, idiopathic pulmonary fibrosis, and peritoneal fibrosis (30, 63, 64); (b) LPA_1_ is an abundantly expressed LPAR in skeletal muscle (42); and (c) pharmacological inhibition (using Ki16425) or genetic deletion of LPA_1_ prevents the fibrotic response induced by LPA intramuscular injection in the tibialis anterior (42). We performed sciatic nerve transection in *Lpar1*-KO mice and collected muscles for analysis 2 weeks later. The results showed lower *Fn1* and *Col1a1* mRNA levels in denervated *Lpar1*-KO mice compared with their WT littermates (Figure 3A). Western blot analyses of muscle homogenates showed that WT mice have elevated levels of fibronectin and CCN2 after denervation compared with *Lpar1*-KO mice (Figure 3, B and C). Consistent with these findings, immunofluorescence and Sirius red staining demonstrated significantly decreased deposition of fibronectin (Figure 3D), CCN2 (Figure 3E), and total collagen (Supplemental Figure 2, C and D) in denervated muscles from *Lpar1*-KO mice. These results indicate that the genetic ablation of *Lpar1* reduces the muscle fibrotic response, suggesting that LPA acts as a fibrogenic factor mediating skeletal muscle fibrosis after denervation.

### LPA signaling is required for the induction of skeletal muscle atrophy markers after denervation.

Denervation by sciatic nerve transection induces severe skeletal muscle atrophy (65). Muscle atrophy is characterized by a reduction in muscle mass resulting from an imbalance between protein synthesis and degradation (66). Two E3 ubiquitin ligases, muscle RING finger 1 (MuRF1) and muscle atrophy F-box (MAFbx)/atrogin-1, have been identified as key regulators of this process. Given that both MuRF1 and atrogin-1 expression are consistently elevated in various forms of muscle atrophy, including denervation (67), we aimed to determine whether LPA signaling is necessary for their induction after denervation.

To investigate this question, we used 2 strategies to disrupt LPA signaling: sciatic nerve transection in (a) WT mice treated with Ki16425 (Supplemental Figure 3, A–D) and (b) *Lpar1*-KO mice (Supplemental Figure 3, E–H). Two weeks after denervation, we observed reduced mRNA expression of MuRF1 and atrogin-1 in the muscles of mice treated with Ki16425 compared with those treated with DMSO (Supplemental Figure 3A). Similarly, *Lpar1*-KO mice exhibited lower mRNA levels of these atrophy markers compared with their WT littermates (Supplemental Figure 3E). Western blot analysis of muscle extracts from GST revealed a lower abundance of MuRF1 in denervated muscles from Ki16425-treated mice compared with controls (Supplemental Figure 3, B and C) and in *Lpar1*-KO mice compared with WT mice (Supplemental Figure 3, F and G). Finally, a key histopathological feature of skeletal muscle atrophy is the reduction in myofiber diameter (8). As expected, 2 weeks after denervation, we observed a significant decrease in fiber size. However, treatment with Ki16425 (Supplemental Figure 3D) or genetic ablation of LPA_1_ in *Lpar1*-KO mice (Supplemental Figure 3H) did not ameliorate muscle fiber atrophy. Nevertheless, in denervated *Lpar1*-KO mice, we found a significant increase in the percentage of small fibers compared with denervated WT mice (Supplemental Figure 3H). Our data demonstrate that blocking LPA signaling decreases atrophy markers but does not prevent myofiber atrophy in muscles subjected to complete denervation for 2 weeks.

### LPA_1_ is required for FAP accumulation after denervation.

**Figure 4:**
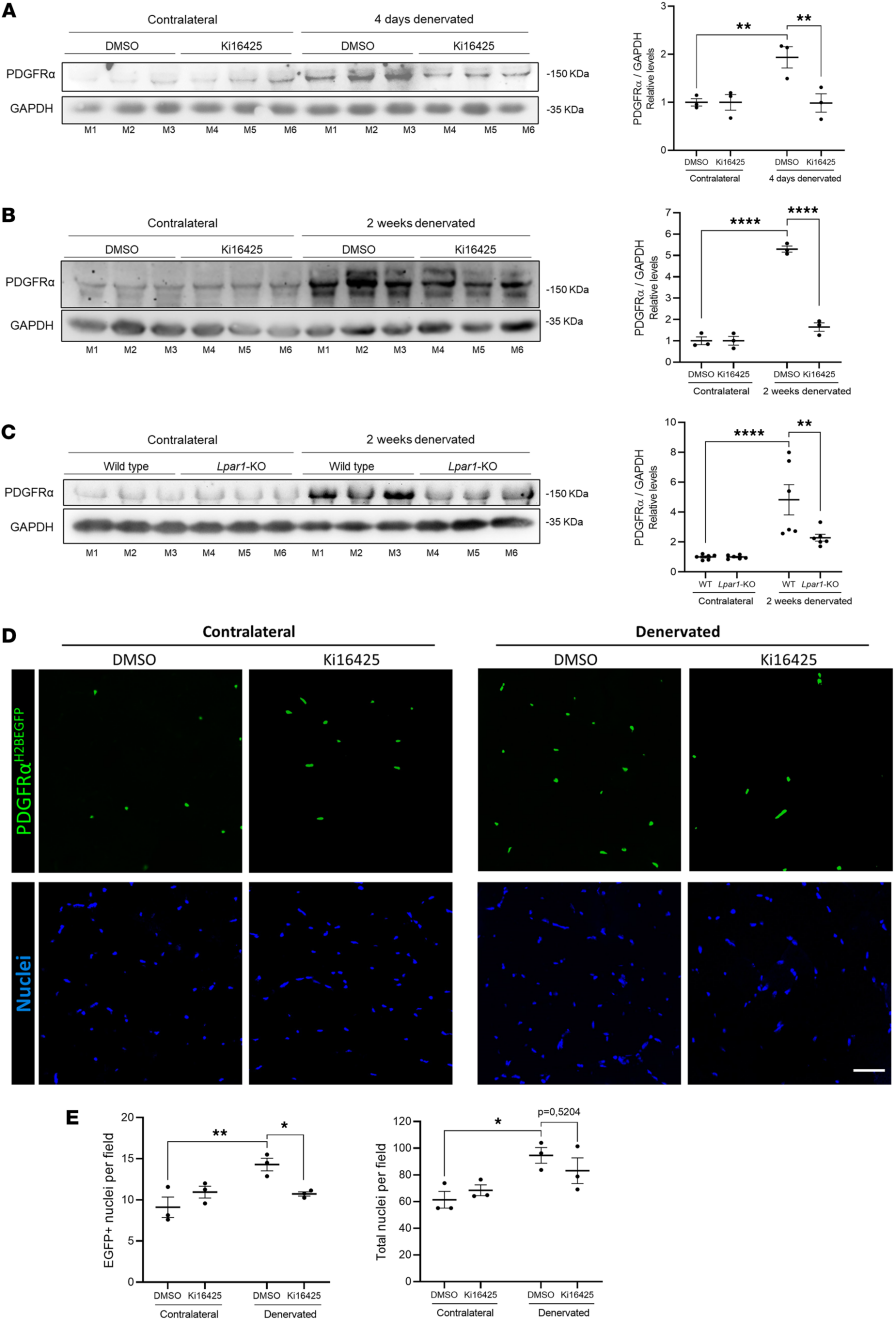
LPA_1_ is required for FAP increase after denervation. (**A**) Immunoblot and quantification of the FAP marker PDGFRα in 6-month-old C57Bl/6J mice 4 days after denervation. Mice were treated with vehicle (DMSO) (*n* = 3) or Ki16425 (*n* = 3) for 3 days before unilateral sciatic denervation. (**B**) DMSO (*n* = 3) or Ki16425 (*n* = 3) was administered intraperitoneally to 6-month-old C57Bl/6J mice for 3 days before denervation, and the treatment was continued daily for 2 weeks. Immunoblot for PDGFRα. (**C**) Six-month-old BALB/c (*n* = 6) and *Lpar1*-KO (*n* = 6) mice were subjected to unilateral sciatic denervation. Western blot analysis of PDGFRα was conducted 2 weeks after denervation. GAPDH was used as the loading control. Levels of PDGFRα were quantified. (**D**) EGFP-positive nuclei in tissue cross-sections from the GST of 6-month-old PDGFRα^H2BEGFP^ knock-in reporter mice treated with DMSO (*n* = 3) or Ki16425 (*n* = 3) for 3 days before unilateral sciatic denervation. (**E**) Quantification of EGFP-positive nuclei and total nuclei per field was performed 4 days after denervation. *P < 0.05, **P < 0.01, ***P < 0.001, ****P < 0.0001, with 1-way ANOVA test. Values are shown as mean ± SEM.

In chronic fibrotic conditions, FAPs are the primary source of myofibroblasts, leading to the intramuscular infiltration of fibrous tissue. Previous studies demonstrated that FAPs are elevated under fibrotic conditions, such as denervation, and colocalize with ECM proteins (13, 14). Given that fibrosis becomes evident by 14 days after denervation and that FAPs are key drivers of initial ECM production, we examined earlier time points to capture the onset of fibrogenic activity. Our current findings showed that the FAP marker PDGFRα was increased 2-fold in the muscles of DMSO-treated mice 4 days after denervation. In contrast, mice treated with Ki16425 displayed reduced PDGFRα levels (Figure 4A). In mice, the DMSO group exhibited a 5-fold increase in PDGFRα protein levels 2 weeks after denervation, whereas the Ki16425-treated group showed basal levels of this protein (Figure 4B). Similarly, denervated *Lpar1*-KO mice also showed reduced PDGFRα levels compared with WT mice (Figure 4C). Additionally, in muscle cross-sections of transgenic mice expressing EGFP in FAP nuclei (PDGFRα^H2BEGFP^), the percentage of FAP nuclei per field was markedly decreased in Ki16425-treated mice compared with DMSO-treated animals after denervation (Figure 4, D and E). These results indicate that LPA/LPA_1_ signaling promotes the accumulation of FAPs in denervated muscle, and its pharmacological or genetic inhibition effectively limits their expansion, suggesting a key role for this axis in driving fibrogenic cell dynamics after nerve injury.

### YAP and TAZ induction after denervation requires the LPA axis.

Next, we focused on the transcription coactivators YAP and TAZ to investigate the signaling pathways involved in the skeletal muscle fibrotic response to denervation mediated by LPARs. Our interest in YAP and TAZ stems from several factors: (a) they act as central integrators of multiple fibrosis-related signaling pathways in various organs (heart, lung, kidney, liver) (47–51); (b) YAP and TAZ are transcriptional coactivators of genes involved in cell proliferation, apoptosis, cell fate, and profibrotic genes such as *Ccn2*, a critical factor involved in neuromuscular disorders (55, 68); (c) mechanical signals and extracellular ligands/growth factors such as LPA can modulate YAP/TAZ activity (69); and (d) we previously showed that YAP and TAZ protein levels are elevated in skeletal muscle after denervation, with YAP accumulating in FAP nuclei (58). Our first approach was to analyze an established YAP/TAZ transcriptional signature (70) in a publicly available RNA-Seq dataset of skeletal muscle from mice subjected to tibial nerve denervation (71). The average expression of all the genes in the YAP/TAZ signature showed increased levels beginning 3 days after denervation (Supplemental Figure 4A).

**Figure 5:**
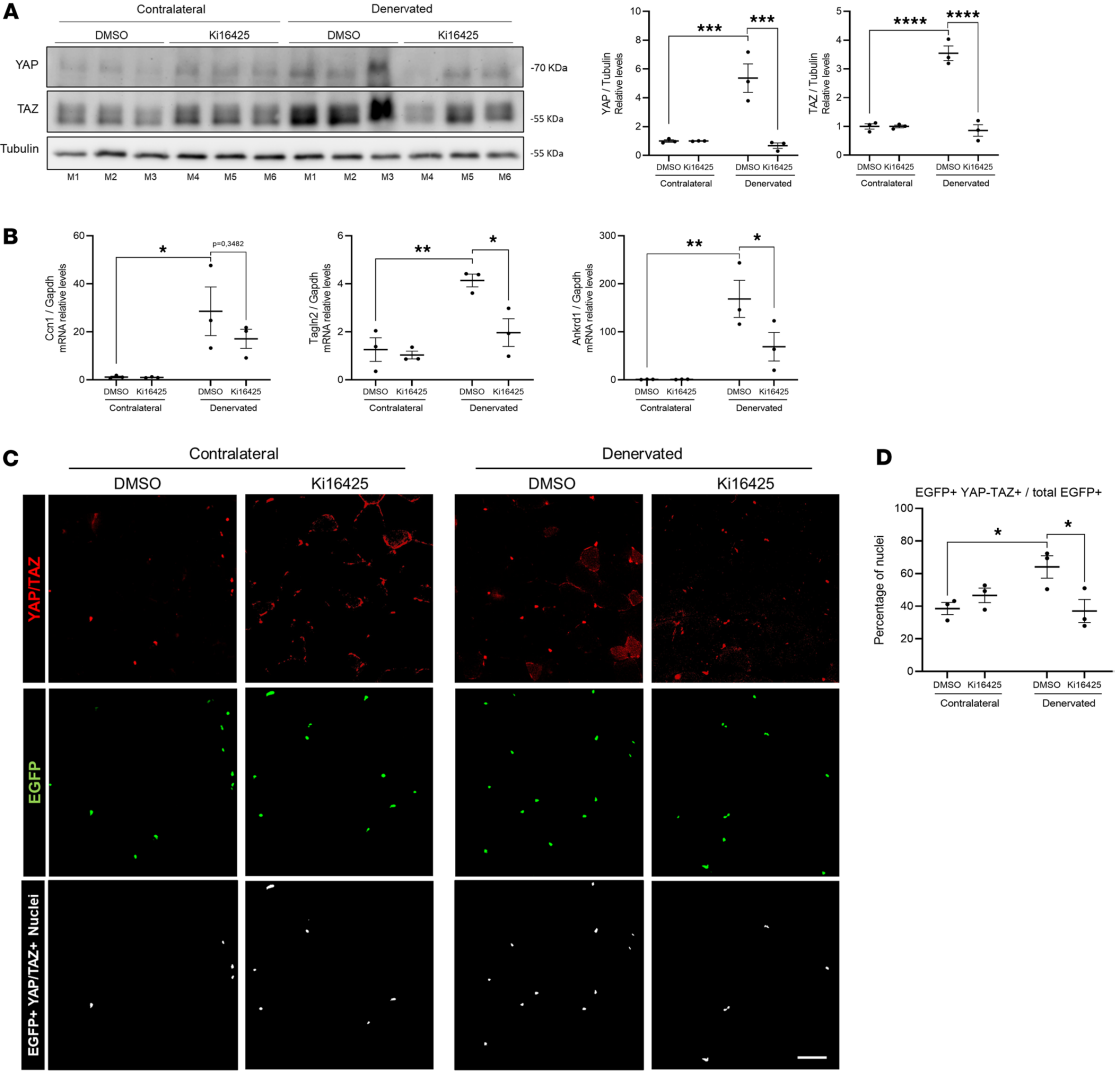
YAP and TAZ induction after denervation requires the LPA axis. (**A**) Western blot and quantification of YAP and TAZ in 6-month-old C57Bl/6J mice treated with vehicle (DMSO) or Ki16425 for 3 days before undergoing unilateral sciatic nerve denervation, with tissues collected 2 weeks after denervation (*n* = 3 per group). GAPDH was used as a loading control. (**B**) Relative mRNA levels of cellular communication network factor 1 (*Ccn1*), transgelin 2 (*Tagln2*), and ankyrin (*Ankrd1*) in GST from mice treated with DMSO or Ki16425, with tissues collected 2 weeks after denervation. (**C**) EGFP-positive nuclei and YAP/TAZ immunofluorescence in tissue cross-sections from the GST of 6-month-old PDGFRα^H2BEGFP^ knock-in reporter mice treated with DMSO (*n* = 3) or Ki16425 (*n* = 3) for 3 days before undergoing unilateral sciatic nerve denervation, with tissues collected 4 days after denervation. The lower panel shows EGFP^+^ YAP/TAZ^+^ nuclei identified by Fiji-based overlap analysis (Binary Feature Extractor, ≥50% overlap threshold), representing the subset of FAP nuclei with nuclear YAP/TAZ localization. (**D**) Quantification of the percentage of FAP nuclei positive for YAP/TAZ. *P < 0.05, **P < 0.01, ***P < 0.001, ****P < 0.0001, with 1-way ANOVA test. Values are shown as mean ± SEM.

We next evaluated YAP and TAZ protein levels in skeletal muscle after denervation and their dependence on LPAR signaling. Using an antibody that recognizes both YAP and TAZ, we found that YAP/TAZ protein levels increased 3- to 6-fold in the denervated limb of mice injected with DMSO. In contrast, mice treated with Ki16425 showed no increase in YAP and TAZ levels in the denervated limb (Figure 5A). Next, we aimed to determine whether YAP/TAZ transcriptional activity is also dependent on LPARs. To do this, we evaluated the expression of classical YAP/TAZ target genes, including cellular communication network factor 1 (*Ccn1*), transgelin 2 (*Tagln2*), and ankyrin (*Ankrd1*), in addition to *Ccn2,* which was already assessed (Figures 2 and 3). Two weeks after denervation, the denervated muscles of DMSO-injected mice showed significantly higher mRNA expression levels of *Tagln2* and *Ankrd1* than those receiving Ki16425 (Figure 5B). In agreement with these findings, *Lpar1*-KO mice exhibited lower YAP and TAZ protein in denervated muscles compared with their WT littermates 2 weeks after denervation (Supplemental Figure 4B). Furthermore, *Lpar1*-KO mice also showed reduced mRNA levels of the YAP/TAZ target genes *Ccn1*, *Tagln2*, and *Ankrd1* (Supplemental Figure 4C).

To determine whether YAP accumulation in FAPs requires LPA signaling, we analyzed the nuclear localization of YAP/TAZ in FAP nuclei using PDGFRα^H2BEGFP^ reporter mice. EGFP-positive nuclei were used to define FAP nuclear regions of interest, and YAP/TAZ nuclear localization was assessed by overlap analysis using Fiji. Four days after denervation, approximately 60% of FAP nuclei (EGFP^+^) showed 50% or greater overlap with YAP/TAZ signal in vehicle-treated mice, whereas this proportion was reduced to 40% in Ki16425-treated mice, comparable to non-denervated muscle (Figure 5, C and D). In summary, our findings indicate that LPA mediates denervation-induced YAP/TAZ expression, transcriptional activity, and nuclear accumulation in FAPs.

### YAP/TAZ activity is required for denervation-induced skeletal muscle fibrosis.

**Figure 6:**
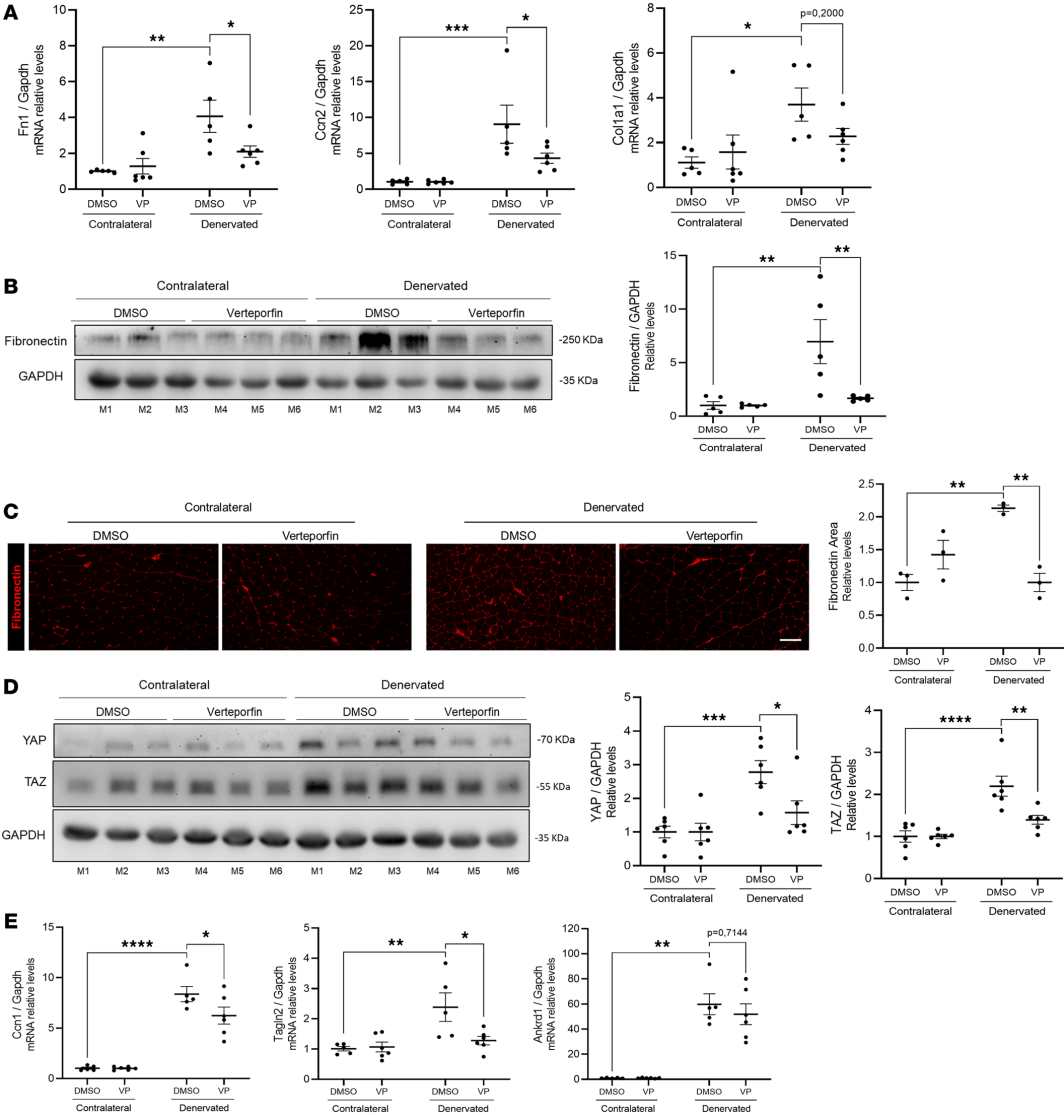
YAP/TAZ activity is required for denervation-induced skeletal muscle fibrosis. Six-month-old C57Bl/6J mice were treated with vehicle (DMSO) (*n* = 5) or verteporfin (*n* = 5) and subjected to unilateral sciatic nerve transection. Skeletal muscles from both hindlimbs were collected 4 days after denervation. (**A**) Fibronectin (*Fn1*), *Ccn2*, and collagen I (*Col1a1*) mRNA levels were measured by RT-qPCR. (**B**) GST homogenates were subjected to SDS-PAGE and immunoblotted for fibronectin. GAPDH was used as a loading control. (**C**) Frozen tissue cross-sections from denervated and contralateral GST were subjected to immunofluorescence for the detection of fibronectin (red). Scale bar: 100 μm. Quantification of fibronectin-positive area. (**D**) Western blot analysis and quantification of YAP and TAZ. (**E**) Relative mRNA levels of *Ccn1*, *Tagln2*, and *Ankrd1*. *P < 0.05, **P < 0.01, ***P < 0.001, ****P < 0.0001 with 1-way ANOVA test. Values are shown as mean ± SEM.

YAP/TAZ has emerged as a promising target in fibrosis because its loss of function or blockade suppresses fibrosis in several organs, as well as myofibroblast differentiation (51, 72, 73). Here, we showed that LPA mediates YAP/TAZ expression, transcriptional activity, and accumulation in FAP nuclei after denervation. To test the relevance of YAP/TAZ activation in denervation-induced fibrosis, we inhibited YAP/TAZ activity using the small-molecule inhibitor verteporfin (74). This inhibitor was administered intraperitoneally to mice subjected to sciatic nerve transection, with daily treatment maintained for 4 days. We found that *Fn1*, *Ccn2*, and *Col1a1* mRNA levels increased in the denervated limb of mice that received the vehicle. However, in mice treated with verteporfin, we found significantly reduced *Fn1* and *Col1a1* (Figure 6A). We also observed reduced accumulation of fibronectin after denervation in mice treated with verteporfin, as determined by Western blot and immunofluorescence analysis, compared with vehicle-treated controls (Figure 6, B and C). Furthermore, denervated muscle from mice treated with verteporfin showed decreased YAP and TAZ levels in the denervated limb compared with vehicle-treated mice (Figure 6D). The mRNA expression of classical YAP/TAZ target genes showed significantly lower levels for *Ccn1* and *Tagln2* in verteporfin-treated mice compared with vehicle-treated controls (Figure 6E). These findings demonstrated that verteporfin treatment effectively reduced the expression of fibrotic markers and YAP/TAZ target genes, along with YAP/TAZ protein levels in denervated skeletal muscle. Altogether, these results indicate that YAP/TAZ activity is required for the development of muscle fibrosis after denervation and validate its pharmacological inhibition as a potential antifibrotic strategy.

### LPA mediates fibrosis and sciatic nerve integrity after transient denervation.

All prior experiments used a model of definitive and complete denervation of the muscle via nerve transection, mimicking severe traumatic injuries. In contrast, nerve crush injuries, defined by the disruption of axonal continuity while preserving the nerve’s connective tissue structures, represent a form of transient denervation that is usually followed by reinnervation of the muscle fibers. Such cycles of denervation and reinnervation can be observed after minor contusions or compressions of peripheral nerves; in diseases like perineural tumor spread (75), ALS (4), myasthenia gravis (76); and in aging (77).

**Figure 7:**
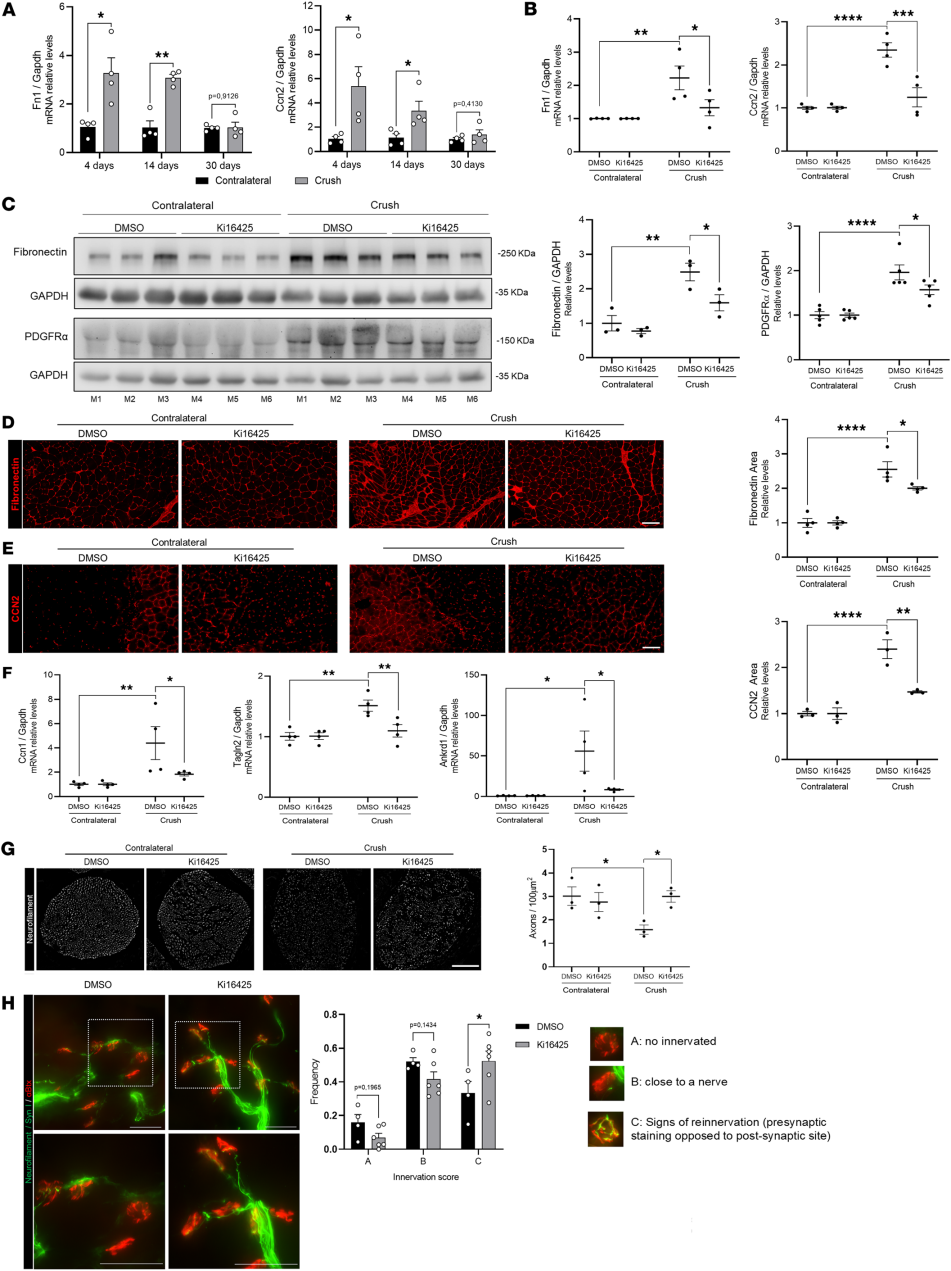
Inhibition of LPA_1_ and LPA_3_ reduces fibrosis and improves sciatic nerve integrity after transient denervation. Three-month-old C57Bl/6J mice were denervated by unilateral crush injury of the sciatic nerve. (**A**) RT-qPCR analysis showing fibronectin (*Fn1*) and *Ccn2* expression in GST from crushed and contralateral control limbs at 4, 14, and 30 days (*n* = 4). *P < 0.05, **P < 0.01, with 2-tailed Student′s *t* test. Values are shown as mean ± SEM. (**B**) Three-month-old C57Bl/6J mice were treated with vehicle (DMSO) (*n* = 4) or Ki16425 (*n* = 4) for 3 days before unilateral sciatic denervation by crush. Skeletal muscles from both hindlimbs were collected 2 weeks after denervation. *Ccn2* and *Fn1* mRNA levels were measured by RT-qPCR. (**C**) GST homogenates were subjected to SDS-PAGE and immunoblot. Levels of fibronectin and PDGFRα were quantified (right). (**D**) Frozen-tissue GST cross-sections from crushed and contralateral controls were subjected to immunofluorescence for the detection of fibronectin (red). Scale bar: 100 μm. Quantification of fibronectin-positive area (right). (**E**) Representative immunofluorescence images of CCN2 (red). Scale bar: 100 μm. Quantification of CCN2-positive area (right). (**F**) RT-qPCR analysis showing *Ccn1*, *Tagln2*, and *Ankrd1* expression in contralateral and crushed GST muscles. (**G**) Representative immunofluorescence images of neurofilament in sciatic nerve showing axonal abundance under different experimental conditions. Scale bar: 100 μm. Quantification of axonal density (axons/100 μm^2^) (right). *P < 0.05, **P < 0.01, ***P < 0.001, ****P < 0.0001 with 1-way ANOVA test. Values are shown as mean ± SEM. (**H**) Representative images of neuromuscular junction staining showing nerves reaching skeletal muscle. For quantification, we classified postsynaptic clusters as non-innervated (a); close to a nerve (b); or with signs of reinnervation, presynaptic staining opposed to postsynaptic site (c). Scale bar: 50 μm. *P < 0.05 with 1-tailed *t* test. Values are shown as mean ± SEM.

To test whether transient denervation induces fibrosis, we performed sciatic nerve crush. Fibrotic markers (fibronectin, CCN2, collagen) increased by days 4–14 and returned to near-baseline levels by day 30 (Figure 7A and Supplemental Figure 5A). YAP/TAZ target genes showed a similar temporal profile (Supplemental Figure 5B). Atrophy markers (atrogin-1, MuRF1) rose on days 4–14 and declined by day 30 (Supplemental Figure 5C), with muscle weight loss and fiber-size variability evident by day 14 (Supplemental Figure 5, D and E). These findings demonstrate that transient denervation triggers a temporary fibrotic response in the skeletal muscle, accompanied by activation of YAP/TAZ target genes and atrophy induction.

Next, we evaluated whether components of the LPA signaling axis were dysregulated in this denervation model induced by nerve crush. We found that *Lpar1*, *Lpar2*, *Lpar3*, and *Lpar6* were upregulated at day 14, returning to near basal levels by day 30 (Supplemental Figure 5F), similar to the pattern observed in the denervation model by nerve transection (Figure 1). Notably, Lpar5 increased uniquely in the crush model. Additionally, ATX exhibited a significant decrease by day 14, which partially recovered by day 30. Altogether, our results indicate a temporal dysregulation of the components of the LPA signaling axis after transient denervation.

We next investigated whether the fibrotic responses in this model of transient denervation were mediated by LPA signaling. Administration of Ki16425 (LPA_1_/LPA_3_ inhibitor) before nerve crush significantly reduced fibronectin, CCN2, and PDGFRα mRNA and protein levels (Figure 7, B and C), as well as fibronectin and CCN2 immunofluorescence signals (Figure 7, D and E). YAP/TAZ targets were also downregulated by Ki16425 in injured mice (Figure 7F). Although atrogin-1 and MuRF1 expression decreased with Ki16425 (Supplemental Figure 3I), muscle fiber diameter was not restored (Supplemental Figure 3J).

This crush model also allowed us to investigate whether inhibiting the LPA signaling axis promotes structural preservation of sciatic nerve and target reinnervation after peripheral nerve injury. Notably, axonal density in the sciatic nerve was preserved in mice treated with Ki16425 compared with vehicle-treated controls at day 14 after crush injury, a time point that reflects ongoing axonal regeneration after the initial degeneration phase (Figure 7G). To determine whether enhanced axonal preservation was associated with improved target reengagement, we quantified the innervation status of postsynaptic acetylcholine receptor clusters labeled with α-bungarotoxin in the GST muscle. Ki16425-mediated LPA antagonism was associated with clearer visualization of nerve fibers reaching skeletal muscle and a shift in the innervation profile, characterized by a reduced proportion of completely denervated endplates and a significant increase in partially reinnervated neuromuscular junctions (Figure 7H).

Altogether, our results demonstrate that the fibrosis induced by transient denervation (evidenced by elevated ECM proteins, PDGFRα levels, and YAP/TAZ target genes), as well as the upregulation of muscle atrophy markers, is mediated by LPA signaling through receptors LPA_1_ and LPA_3_. Notably, these effects recapitulate the fibrotic and signaling responses observed after complete sciatic nerve transection, reinforcing the notion that LPA signaling is a key driver of muscle fibrosis regardless of the severity of sciatic nerve damage. These findings also suggest a potential role for components of the LPA axis not only in regulating muscle fibrosis but also in facilitating axonal regeneration and the ability of axons to reach and reoccupy the motor endplate, providing a structural correlate for potential improvements in functional recovery.

## Discussion

Fibrosis is a hallmark of several chronic, inflammatory, and autoimmune pathologies affecting almost every organ. Previous studies by us and other laboratories have revealed the involvement of numerous molecules in the pathology of skeletal muscle fibrosis (78–80). The inhibition of pathways that drive fibrosis induction has consistently resulted in improved muscle contractility, strength, and overall tissue function (21, 81). Therefore, a better understanding of the molecular mechanisms that modulate fibrosis is essential for the development of effective therapies that target the underlying causes of chronic diseases, including fibrotic neuromuscular disorders.

Our laboratory has been interested in studying the profibrotic effects of LPA in skeletal muscle (42, 56, 57, 82). Previous work has shown that exogenous LPA administration mimics key features of muscle fibrosis, primarily through LPA_1_, leading to ECM accumulation and FAP expansion (42). More recent studies described the importance of the LPA/LPA_1_ axis in regulating FAP responses, specifically CTGF/CCN2 expression, proliferation, differentiation into myofibroblasts, and migration (56, 57, 82). Our current findings provide a comprehensive description of the LPA signaling pathway as a driver of muscle fibrosis after peripheral nerve injury.

First, our results revealed an early dysregulation of key components of the LPA signaling axis in skeletal muscle. This included increased expression of several LPA receptors, alterations in enzymes involved in LPA metabolism, and a transient early increase in intramuscular LPA levels, indicating that denervation is associated with rapid engagement of the LPA signaling pathway at multiple regulatory levels. A similar pattern of LPA axis dysregulation was reported in a murine model of δ-sarcoglycanopathy, a chronic muscle disease characterized by progressive fibrosis, where we reported increased expression of LPA_1_ and LPA_6_, reduced ATX levels, and elevated intramuscular LPA concentrations (83). Likewise, in renal fibrosis, LPA_1_ expression is significantly upregulated in the kidneys of mice subjected to unilateral ureteral obstruction (63). Together, these observations suggest that remodeling of the LPA axis may represent a conserved feature of fibrotic pathologies across tissues.

Notably, a trend toward increased LPA levels was also detected in contralateral muscles after unilateral denervation, raising the possibility of systemic or cross-limb effects. Similar crossover responses have been reported in denervation and unilateral injury models, in which contralateral muscles exhibit alterations in proteolytic activity, inflammation, and fibrotic remodeling in the absence of direct injury (84, 85). These findings indicate that unilateral denervation can elicit broader systemic or neural-mediated responses that may influence muscle homeostasis beyond the injured limb. Importantly, the transient increase in intramuscular LPA levels, together with sustained dysregulation of LPA receptors and metabolic enzymes, supports the interpretation that denervation-induced fibrosis is primarily associated with persistent remodeling of the LPA signaling axis rather than prolonged elevation of LPA levels alone.

In this context, although our data demonstrate that denervation induces coordinated changes in multiple components of the LPA signaling axis in vivo, the specific cellular sources responsible for LPA production and the cell type–specific regulation of LPA receptor expression and lipid metabolism within denervated muscle remain to be fully defined. Elucidating these aspects will be important to clarify the molecular mechanisms by which denervation engages LPA signaling and to determine the spatial and temporal organization of this pathway during muscle remodeling.

Collectively, this evidence suggests that the LPA signaling axis may play an important role in driving fibrosis after denervation. This hypothesis is further supported by our current results showing that denervation-induced muscle fibrosis is significantly reduced in mice treated with the LPA_1_/LPA_3_ receptor antagonist Ki16425 and in *Lpar1*-KO mice. Comparable antifibrotic effects of LPAR inhibition have also been documented in models of dermal, renal, and pulmonary fibrosis (35, 36, 38). These findings also support the idea of targeting other components of the LPA pathway, such as the enzyme ATX, which has been associated with antifibrotic effects in preclinical models (37, 39). Furthermore, the development of specific LPA_6_ antagonists may help to directly test its functional role, given that LPA_6_ is one of the most abundantly expressed receptors in skeletal muscle and is upregulated in other fibrotic muscle conditions (86).

The LPA signaling axis has been linked to the regulation of myofiber size in various injury models. In a regeneration context, the intramuscular injection of LPA promoted hypertrophic responses in a cardiotoxin-induced muscle injury model (87). In contrast, other studies have suggested that LPA may play a role in muscle atrophy. Puigdomenech-Poch et al. showed that *Lpar2* deficiency protected against muscle atrophy in SOD1^G93A^ mice (88), and Davies et al. found that intraperitoneal injection of LPA significantly worsened muscle atrophy after massive rotator cuff tears in rats (41). These inconsistent results suggest that the effects of LPA on myofiber size may be context dependent, promoting hypertrophy during regeneration while contributing to atrophy in chronic injury or degenerative disease models. In our study, interfering with LPA signaling reduced the expression of atrophy-related genes, supporting the hypothesis that LPA contributes to muscle atrophy in chronic injury contexts. However, this transcriptional shift was not accompanied by a restoration of myofiber cross-sectional area. This lack of structural recovery may be explained by the strong dependence of myofiber size on stable and functional reinnervation. Although we observed increased axonal density and a higher proportion of early reinnervation events, the maturation of fully functional neuromuscular junctions is a prolonged process that extends beyond the 14-day observation period (89, 90). Consequently, reductions in atrophy markers may precede measurable myofiber hypertrophy. Further studies at extended time points will be required to define the temporal dynamics of LPA signaling during muscle remodeling and to assess its long-term impact on trophic recovery. In addition, the involvement of other LPA receptors in the regulation of muscle atrophy and remodeling cannot be excluded, and such contributions may account for LPA-dependent effects not fully addressed by targeting LPA_1_ alone.

In skeletal muscle, FAPs are the main source of myofibroblasts during fibrosis. Elevated numbers of FAPs have been reported in chronic fibrotic conditions, including in patients with DMD (17), *mdx* mice (13), and ALS transgenic mice hSOD1^G93A^ (18). Consistent with earlier studies (13, 14), we found that FAPs accumulate in denervated muscle. Notably, when LPA/LPA_1_ signaling was pharmacologically or genetically inhibited, we observed reduced PDGFRα protein levels and fewer EGFP-positive cells in PDGFRα^H2B-EGFP^ knock-in reporter mice, aligning with the fact that LPA promotes FAPs’ proliferation both in vitro and in vivo in skeletal muscle (42, 56). These data support a role for the LPA signaling axis in fibrosis by promoting FAP activation. However, this result does not exclude the possibility that other resident or infiltrating cell types in skeletal muscle may also contribute to the fibrotic response mediated by LPA, a premise that requires additional investigation.

Denervation leads to increased YAP expression, transcriptional activity, and nuclear accumulation in FAPs (58). Our study demonstrates that LPA mediates those changes through LPA_1_ signaling. YAP/TAZ have been identified as downstream effectors of LPA signaling in multiple cellular contexts (44–46), including the mediation of LPA-induced FAP activation and migration in vitro (56, 57). Our data indicate that YAP/TAZ activity is required for the development of skeletal muscle fibrosis after denervation. Furthermore, YAP/TAZ has been implicated in fibrosis in various organs, including the liver, lung, and kidney (47–51), as well as in a model of acute muscle injury (59). Since LPA signaling contributes to fibrosis in these tissues (35, 37–41), the results suggest that YAP/TAZ may serve as a common downstream effector coordinating LPA-driven fibrotic responses.

Transient denervation by sciatic nerve crush induced a reversible fibrotic response in skeletal muscle, accompanied by YAP/TAZ activation and increased expression of atrophy markers. The pharmacological inhibition of LPA_1_/LPA_3_ with Ki16425 attenuated fibrosis, YAP/TAZ, and atrophy-related genes after transient denervation. These results align with those observed in Ki16425-treated mice and *Lpar1*-KO mice under denervation by nerve transection, indicating that targeting this pathway can modulate muscle fibrosis in both transient and definitive denervation.

Moreover, our results also suggest that the LPA signaling axis may play a role in preserving sciatic nerve integrity after injury, as the analysis of nerve damage following crush showed higher axonal density in mice treated with Ki16425 compared with vehicle-treated controls. Although this finding may reflect enhanced axonal regeneration, it could also result from a prolonged inhibition of degeneration or the combined effects of both processes. This aligns with previous work in ALS transgenic mice (hSOD1^G93A^), where disrupting LPA_1_ slows down disease progression by delaying motoneuron degeneration, improving motor function, and extending the survival of ALS mice (91). Additional evidence supports the role of LPA signaling in peripheral nerve pathology. For instance, Szepanowski et al. reported that the immunomodulatory drug fingolimod, which inhibits ATX and thereby reduces LPA biosynthesis, improves peripheral nerve regeneration (92). In another study, treatment with the LPA_1_ receptor antagonist AM095 increased the number of large-caliber myelinated axons in a rat model of experimental autoimmune neuritis (a Guillain-Barré syndrome model) (93). Additionally, Kheyrollah et al. proposed that *Lpar2* knockdown could enhance corneal nerve regeneration (94). In the CNS, intrathecal injection of lysophosphatidylcholine (precursor of LPA) induced potent dorsal root demyelination, which was markedly attenuated or abolished in *Enpp2*^+^/^–^ or *Lpar1*-KO mice (95). These findings further strengthen the argument that inhibiting LPA signaling confers neuroprotective and regenerative benefits, making it a highly attractive therapeutic target.

Notably, inhibitors of ATX and LPARs, such as GLPG1690, BMS-986020, and SAR100842, have advanced into clinical trials for fibrotic diseases including idiopathic pulmonary fibrosis and systemic sclerosis (96). In a phase 2 trial, the oral LPA_1_ antagonist BMS-986020 reduced plasma LPA levels, slowed lung function decline, and was well tolerated in patients with idiopathic pulmonary fibrosis and progressive pulmonary fibrosis, supporting its progression to phase 3 studies (97). These promising results demonstrate that LPA signaling can be safely inhibited in humans and highlight its potential for therapeutic application. Thus, pharmacological inhibition of LPA signaling may offer dual benefits in neuromuscular diseases by reducing muscle fibrosis and enhancing axonal regeneration. This therapeutic strategy could be particularly valuable in conditions characterized by neuromuscular junction disruption, such as ALS, myasthenia gravis, DMD, sarcoglycanopathies, age-related neuromuscular deterioration, and peripheral nerve damage caused by trauma or accidents. This represents a compelling next step, offering a rational basis for future preclinical and clinical studies.

The dual impact of LPA signaling inhibition on muscle and nerve integrity parallels previous findings from our lab in ALS transgenic mice (hSOD1^G93A^), where blocking CCN2 reduced skeletal muscle fibrosis, mitigated myelin degeneration in the sciatic nerve, and improved neuromuscular junction innervation (4). LPA can induce CCN2 skeletal muscle and FAPs (42, 56), and it is a well-established transcriptional target of YAP/TAZ (55, 68). Moreover, YAP binding to TEAD transcription factors is essential for LPA-mediated CCN2 induction in fibroblasts (57). Collectively, these observations suggest that CCN2 could mediate LPA/LPA_1_/YAP/TAZ responses in both muscle and peripheral nerves.

Overall, the data presented in this study indicate that the LPA/LPA_1_/YAP/TAZ signaling axis is a key driver of muscle fibrosis and nerve integrity after peripheral nerve injury. A deeper understanding of this pathway’s role in neuromuscular disorders characterized by muscle fibrosis and loss of innervation may help identify shared therapeutic targets. Modulating this signaling axis could represent a promising strategy to mitigate fibrosis and preserve neuromuscular integrity, thereby improving patients’ quality of life.

## Methods

### Sex as a biological variable.

In all studies, both male and female mice were used. Sex was not analyzed as an independent biological variable, and data were not stratified by sex.

### Animals.

C57Bl/6J (strain 000664) and Pdgfra^tm11(EGFP)Sor^ mice (referred to as PDGFRα^H2BEGFP^) (strain 007669) were obtained from The Jackson Laboratory. The *Lpar1*-KO mice were originally generated and characterized by Chun et al. (98). *Lpar1*-KO mice were maintained on a BALB/c background. All studies were conducted in 3- to 6-month-old male and female mice. Mice were maintained in 12-hour light/12-hour dark cycles and fed ad libitum with a standard chow diet in the animal facility (Laboratorio de Farmacología Preclínica PACIE USS) at the Fundación Ciencia & Vida, Universidad San Sebastián.

### Denervation and crush surgery.

Before the surgery itself, aseptic techniques were performed, such as cleaning the area with chlorhexidine 2%. Mice were anesthetized with isoflurane delivered via a precision vaporizer (induction at 3%–4% and maintenance at 1%–2% in oxygen) and positioned on a heating pad to maintain body temperature throughout the procedure. The surgeries consisted of exposing the sciatic nerve through an incision at the level of the mouse’s hip. After the nerve was exposed, for denervation surgery, a 2–5 mm of the sciatic nerve was unilaterally transected only in the right hindlimb to restrict reinnervation (8); for crush surgery, a sciatic nerve region was crushed 3 times perpendicular to the nerve using an ultrathin tweezer (504506, WPI) embedded with graphite powder, and each crushing was performed orthogonally for 5 seconds, so the crush area was as wide as the tweezer tip (1 mm approximately) (99). Sham surgery was an identical procedure on the contralateral posterior left limb, except for nerve damage. Subsequently, the wound was closed with a 9 mm autoclip (205016, Mikron). Postoperative analgesia was provided with tramadol (30 mg/kg) for 3 consecutive days. Then, 4, 14, or 30 days after surgery, mice were anesthetized with isoflurane and euthanized by cervical dislocation. GST muscles were dissected from both hindlimbs. Muscle samples for cryosectioning were frozen in liquid nitrogen–cooled isopentane and stored at –80°C until processing. Sciatic nerves were excised from mice and immediately fixed for 1 hour by immersion in 4% paraformaldehyde (in 1× PBS, pH 7.2) at room temperature.

### Drug treatment.

The LPAR inhibitor Ki16425 was obtained from Cayman Chemical (355025-24-0) and resuspended in sterile DMSO. A 5 mg/kg dose was administered daily by intraperitoneal injection to 3- to 6-month-old WT mice. This dosing regimen was selected based on previous in vivo studies, including our own work, in which Ki16425 effectively inhibited LPA_1_-dependent signaling without reported overt toxicity (42, 100, 101). Denervation or crush surgery was performed 3 days after the first injection, and muscles were collected 4 and 14 days after surgery (2). Inhibition of YAP/TAZ was assessed by disrupting its activity with verteporfin (Sigma-Aldrich, SML0534). Verteporfin was resuspended in sterile DMSO, and mice received 50 mg/kg intraperitoneally (59) every day (4 doses), beginning on the same day of denervation surgery.

Throughout the treatment periods, mice were monitored daily for body weight, grooming behavior, posture, and overall activity, and no signs of distress or adverse effects were observed.

### Western blot analysis.

Muscles were homogenized in 10 volumes of Tris-EDTA buffer pH 7.4 with a mixture of protease/phosphatase inhibitors and 1 mM PMSF using an Ultra-Turrax T25 Basic at 22,000 rpm (Kinematica). Then, homogenized tissue was mixed with an equal volume of a solution containing 20% glycerol, 4% SDS, and 0.125 M Tris pH 6.8; heated (50°C for 20 min), and spun down (16,400 g for 10 min) to pellet insoluble material. According to the manufacturer’s instructions, the total protein concentration was determined using the Micro BCA Protein assay kit (23235, Thermo Fisher Scientific).

Next, 40 μg of protein extracts were subjected to SDS-PAGE and transferred onto PVDF membranes (88518, Thermo Fisher Scientific). Membranes were blocked with 5% nonfat milk in TBS-Tween (50 mM Tris-Cl, pH 7.6; 150 mM NaCl; 0.1% Tween 20) and probed with the following antibodies at 4°C overnight: anti-CCN2 (sc-14939, Santa Cruz Biotechnology), anti-fibronectin (F3648, Sigma-Aldrich), anti-PDGFRα (AF1062, R&D Systems), anti-YAP/TAZ (8418S, Cell Signaling Technology), anti-autotaxin (10005375, Cayman Chemical), anti-GAPDH (631402, BioLegend), anti-Murf1 (C-11: sc-398608, Santa Cruz Biotechnology), and anti-tubulin (T5168, Sigma-Aldrich). Primary antibodies were detected with HRP-conjugated secondary antibodies: anti-rabbit IgG (31450, Invitrogen), anti-mouse IgG (31430, Invitrogen), and anti-goat IgG (sc-2020, Santa Cruz Biotechnology). All immunoreactions were visualized by enhanced chemiluminescence (Pierce) using a ChemiDoc-It HR 410 imaging system (Upland). Densitometric quantification was performed using Image Studio Lite software version 5.2 (LI-COR Biosciences).

### RNA isolation, reverse transcription, and qPCR.

Total RNA was isolated from GST using TRIzol reagent (Invitrogen) according to the manufacturer’s instructions. cDNA synthesis was performed using random primers and M-MLV (Invitrogen). Quantitative real-time PCR (qPCR) was performed on an Eco Real-Time PCR System (Illumina) using PowerUp SYBR Green Master Mix (Applied Biosystems) and primer sets (See Supplemental Table 1). mRNA expression was quantified using the comparative ΔCt method (2^-ΔΔCT^), using Gapdh as the reference gene. The mRNA levels were expressed relative to the mean expression in the control condition.

### Muscle indirect immunofluorescence and microscopy.

Tissue cross-sections were fixed for 10 minutes in 4% paraformaldehyde, washed in 1× PBS, and permeabilized in 1% Triton X-100 in 1× PBS. Samples were blocked for 60 minutes in 1% BSA in 1× PBS and incubated overnight at 4°C with primary antibodies: anti-CCN2 (1:50; D8Z8U, Cell Signaling Technology), anti-laminin (1:200; L0663, Sigma-Aldrich), anti-fibronectin (1:200; F3648, Sigma-Aldrich), and anti-YAP/TAZ (1:200; 8418S, Cell Signaling Technology). Samples were then washed in 1× PBS and incubated for 1 hour at room temperature with a secondary antibody, Alexa Fluor 568 donkey anti-rabbit IgG (H+L) (1:500; A10042, Invitrogen) or Alexa Fluor 488 goat anti-rat IgG (H+L) (1:500; A11006, Invitrogen), and washed in 1× PBS. Then, the samples were incubated with Hoechst 33342 (2 mg/mL diluted in 1× PBS) for 10 minutes and mounted with a fluorescent mounting medium (DAKO, Sigma-Aldrich). Muscle samples were imaged on a Zeiss Axioscope fluorescence microscope (equipped with an AxioCam 202 mono and Colibri 3 LED illumination) (Figure 2, D and E, Figure 3, D and E, Figure 6C, and Figure 7, D and E), an Olympus FV1200 confocal microscope (Figure 4D, Figure 5C, and Figure 7 G, and H), and a Leica 218 DMi8 microscope (Supplemental Figure 2).

### Neuromuscular junction evaluation.

Longitudinal 40 μm cryosections of fresh frozen GST muscle were collected on 24-well multiplates containing 4% paraformaldehyde and fixed for 2 hours. The sections were washed in PBS and blocked/permeabilized overnight with 1% BSA, 1% jellyfish, and 0.25% Triton X-100 in 1× PBS. Samples were incubated with α-bungarotoxin (1:500, Life Technologies), mouse anti-neurofilament coupled to Alexa Fluor 647 (1:500; 837709, BioLegend), and rabbit anti-SynI (1:250; 64581, Abcam) for 2 hours at room temperature. After 3 washes with PBS, samples were incubated with Alexa Fluor 647 secondary anti-rabbit antibody (1:500; A21244, Invitrogen). After 3 washes with 1× PBS, samples were carefully mounted on glass slides with a fluorescent mounting medium and observed on a Leica Sp8 confocal microscope. Next, 60–100 α-bungarotoxin clusters were counted per mice, and we categorized the neuromuscular junctions based on the degree of axonal contact: denervated (stage A), in proximity to a nerve (stage B), and showing some degree of reinnervation observed by presynaptic staining opposing α-bungarotoxin clusters (stage C).

### Determination of fiber diameter.

Fiber size was calculated on reconstructed images of each GST muscle from laminin immunofluorescence. Muscle fiber segmentation was performed using Cellpose 2.0 with the cyto2 model (102), which outputs labeled regions of interest (ROIs). These ROIs were further processed in Fiji/ImageJ (NIH) using the Label to ROI plugin (103), which allowed correction of area bias and generation of binary masks.

### Sirius red staining.

Total collagen content was detected by staining with 0.1% Sirius red in picric acid (104). Briefly, GST cryosections were fixed for 30 minutes in precooled 100% ethanol at –20°C and washed for 3 minutes in distilled H20. The slides were incubated in saturated picric acid at 60°C for 1 hour, washed for 3 minutes in distilled H20, set for 2 minutes at room temperature in a 0.2% solution of phosphomolybdic acid, and washed for 3 minutes in dH20. Then, the samples were incubated in saturated picric acid with 0.1% Sirius red (365548, direct red 80 dye; Sigma-Aldrich) for 5 minutes at room temperature and washed in 0.001 N hydrochloric acid for 2 minutes. Next, slides were rapidly dehydrated through graded alcohols starting at 70%, then to xylene, and finally cover-slipped with Entellan (107961; Sigma-Aldrich). Sections were imaged with a Leica DMi8 microscope.

### Muscle image analysis.

Image processing and analysis of muscle samples were performed using Fiji (v1.53f51, ImageJ). Data were expressed relative to the contralateral limb. Macros were created and run in batch mode without user interaction once the parameters were optimized using training images. The quantification of CCN2 and fibronectin area was calculated as the percentage of pixels above a given threshold. The percentage of FAPs was determined by counting the total number of nuclei (stained with Hoechst) and nuclei positive for EGFP (FAPs) per field. Nuclei were counted using the analyze particle function after establishing a threshold to distinguish the background from the particles of interest. For total nuclei counting, the threshold was set to detect all nuclei; for detecting FAP nuclei, the threshold was adjusted to identify only EGFP-positive nuclei. The percentage of FAP nuclei positive for YAP/TAZ was determined with the tool Binary Feature Extractor (Plugins/BioVoxxel). Using FAP nuclei ROI as the “Objects image,” YAP/TAZ fluorescence as the “Selector image,” and 50 as the “Overlap in percent” (minimal overlap fraction of the selector area with the object area). This method allowed us to identify the number of FAP nuclei with an overlap of 50% or greater for YAP/TAZ staining as EGFP^+^ YAP/TAZ^+^ (Figure 5C).

### LPA ELISA kit.

LPA levels in GST muscle were quantified using a competitive ELISA kit, following the manufacturer’s instructions (MBS2700658, MyBioSource). Briefly, whole GST muscles were homogenized in 1× PBS and centrifuged at 12,000*g* for 5 minutes at 4°C. Protein concentration in the resulting supernatant was determined, and equal amounts of total protein were used for LPA quantification.

### Transcriptomics analyses.

Bulk RNA-Seq data from control and denervated muscles were obtained from the NCBI’s Sequence Read Archive (SRA, accession SRP196460) (71). Raw data processing was performed as previously described in Gallardo et al. (58). Gene signatures YAP/TAZ was retrieved from Cordenonsi et al. (70). Signature scores were calculated as the average of the expression values of all genes included in each signature.

### Histological processing of nerves.

After fixation, sciatic nerves were washed 3 times in 1× PBS for 10 minutes each. Then, the nerves were dehydrated by consecutive immersion in 5%, 10%, and 20% sucrose (in 1× PBS) for 1 hour each at room temperature. Next, the nerves were immersed in a homogeneous mixture of 20% sucrose and OCT (Tissue-Tek) (1:1) for 1 hour, followed by immersion in OCT for an additional hour at room temperature. Finally, the nerves were embedded in OCT, rapidly frozen on dry ice, and stored at –80°C. Transversal sections of 10 μm thickness were produced from the nerve OCT blocks using a cryostat. These sections were mounted on silane-coated slides and stored at –20°C.

### Sciatic nerve immunofluorescence and microscopy.

Sciatic nerve sections were treated with a permeabilizing and blocking solution (0.1% Triton X-100, 5% gelatin from cold water fish skin in 1× PBS) for 1 hour. Following this step, the sections were incubated overnight at 4°C with an anti-neurofilament 200 antibody (1:1,000; N4142, Sigma-Aldrich). The next day, samples were washed 3 times in 1× PBS and incubated for 2 hours at room temperature with Alexa Fluor 488 chicken anti-rabbit IgG (H+L) (1:500; A21441, Invitrogen). After this incubation, the sections were washed again 3 times in 1× PBS and mounted with DAKO mounting media containing DAPI (1:3,000). The mounted sections were stored at room temperature, protected from light. Single-plane confocal images were acquired using an OLYMPUS FluoView 1200 confocal microscope with a 40× objective (NA 1.3) and a pinhole diameter of 300 μm.

### Axonal density quantification.

Image processing and analysis were carried out using Fiji software (NIH). To reduce background noise, a Gaussian-blurred duplicate of each image (radius = 25) was subtracted from the original. The resulting denoised images were then thresholded by intensity and converted to binary. Binary particles corresponding to axons were filtered based on size and automatically counted for each field. The total nerve area was manually delineated by tracing the epineurium boundary in each image. Axonal density was calculated as the number of axonal particles divided by the corresponding nerve area.

### Statistics.

All results are presented as the mean ± SEM. Statistical analyses were performed using GraphPad Prism 8. Differences were assessed using an unpaired 2-tailed Student′s *t* test for 2 comparisons, and 1-way ANOVA and Bonferroni’s post hoc test for multiple comparisons. A difference was considered statistically significant with a *P* value of 0.05 or less. The number of biological replicates performed is indicated in each figure. Specific values are indicated by dots.

### Study approval.

The experimental animal protocols were approved by the Animal Ethics Committee of the Fundación Ciencia & Vida, Universidad San Sebastián, Providencia, Chile (approval P076/2024). All experiments were performed in accordance with the institutional guidelines and regulations.

### Data availability.

Values for all data points in graphs are reported in the Supporting Data Values file. Additional data related to this study are available from the corresponding author upon reasonable request.

## Author contributions

MCS, ACC, JCC, SM, and EB conceptualized the study. MCS, ACC, JFC, NWM, SBA, FVR, DLR, and CGR conducted experiments and analyzed results. FSG performed bioinformatic analyses. EB, FAC, CPV, and JC provided reagents. MCS wrote the original draft of the manuscript and generated all figures. All authors reviewed and edited the manuscript. Co-first authorship order was randomly determined.

## Conflict of interest

JC has an employment relationship with Neurocrine Biosciences, a company that may potentially benefit from the research results. JC’s relationship with Neurocrine Biosciences has been reviewed and approved by Sanford Burnham Prebys Medical Discovery Institute in accordance with its conflict-of-interest policies.

## Funding support

- FONDECYT 1190144 and 1230054 grants (to EB).
- CARE-AFB170005 grant (to EB).
- Centro Científico y Tecnológico de Excelencia Ciencia & Vida Basal FB210008 grant (to EB and DLR).
- PhD Scholarship from the Chilean National Agency for Research and Development (ANID; 21241714) (to JFC).

## Supplementary Material

Supplemental data

Unedited blot and gel images

Supporting data values

## Version 1 04/22/2026

Electronic publication
