# Supplementary material for: Lysophosphatidic acid mediates skeletal muscle fibrosis in denervation via activation of YAP/TAZ
Source: JCI Insight. 2026 Apr 22;11(8):e198388. doi: 10.1172/jci.insight.198388 (PMC13135410; doi:10.1172/jci.insight.198388)
Supplement: Supplemental data [file jciinsight-11-198388-s117.pdf]

Gene	Forward Sequence (5’-3’)	Reverse Sequence (5’-3’)
Lpar1	CTGCCTCTACTTCCAGCCCTGTAA	TGCTCACTGTGTTCCATTCTGTGG
Lpar2	GCTGGTTATTGCAGCCATCG	ACACCCACGATGAGTGTGAC
Lpar3	CCACTTTCCCTTCTACTACCTGCT	GACGGTCAACGTTTTCGACACC
Lpar4	GTCAACAATGCGACCACCAC	CTTGCGGAGGGTTCTAAGCA
Lpar5	ACTCCACGCTGGCTGTATATG	GTAGCCAAAGGCCTGGTATTC
Lpar6	GATCACTCTCTGCATCGCTGTTTC	CCCTGAACTTCAGAGAACCTGGAG
Plpp1	GGGAGACTGGGCAAGACTCTT	CACTCGAGAAAGGCCCACAT
Plpp2	CGCGATCCAACTTCAACAACT	CAGCCCCGAACAGAAAGGT
Plpp3	CCATCCTGGCGATCATTACAG	AAAGGAAGCATCCCACTTGCT
Enpp2	GACCCTAAAGCCATTATTGCTAA	GGGAAGGTGCT GTTTCATGT
Ctgf/ Ccn2	CAGGCTGGAGAAGCAGAGTCGT	CTGGTGCAGCCAGAAAGCTCAA
Fn1	AGATTGGCGACAAGTGGAGG	AGGTTTGCAGGTCCATTCCC
Col1a1	GGTATGCTTGATCTGTATCTGC	AGTCCAGTTCTTCATTGCATT
Ccn1	TAAGGTCTGCGCTAAACAACTC	CAGATCCCTTTCAGAGCGGT
Tagln2	AGCAGATCCTCATCCAGTGG	CCATCTGCTTGAAGGCCA
Ankrd1	GGATGTGCCGAGGTTTCTGAA	GTCCGTTTATACTCACAGAC
Trim63	GCTGGTGGAAAACATCATTGACAT	CATCGGGTGGCTGCCTTT
Fbxo32	GCA AACACTGCCACATTCTCTC	CTTGAGGGGAAAGTGAGACG
Gapdh	TGATGACATCAAGAAGGTGGTAAG	TCCTTGGAGGCCATGTAGGCCAT

Supplementary Table 1. RT-qPCR primers list used in this work.

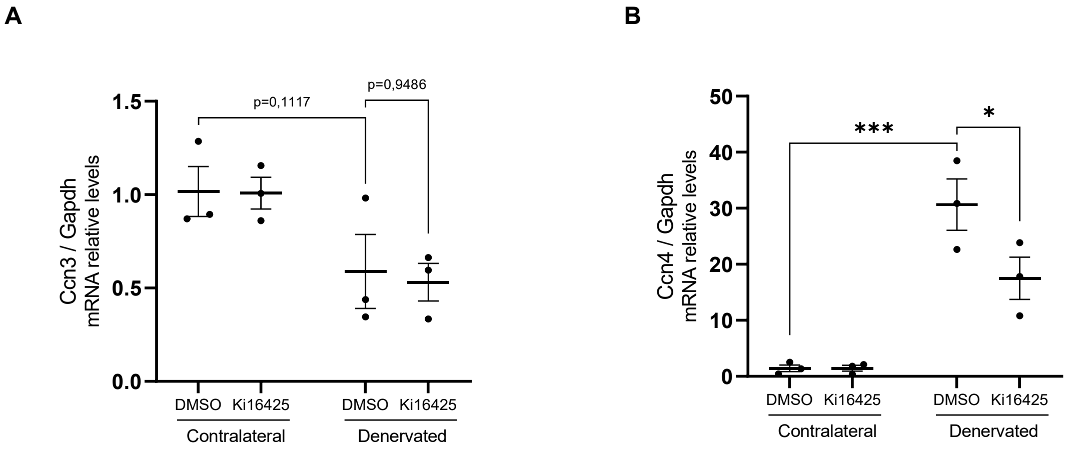

Supplementary Figure 1: Pharmacological inhibition of LPA_1 and LPA_3 reduces the Ccn4 levels after denervation. The vehicle (DMSO) (n=3) or Ki16425 (n=3) was administered intraperitoneally to 6-month-old C57Bl/6J mice for three days before denervation and maintained the treatment daily for 2 weeks. *Ccn3* (**A**) and *Ccn4* (**B**) mRNA levels were measured by RT-qPCR. ***P < 0.001, *P < 0.05 with One-way ANOVA test. Values represent means ± SEM.

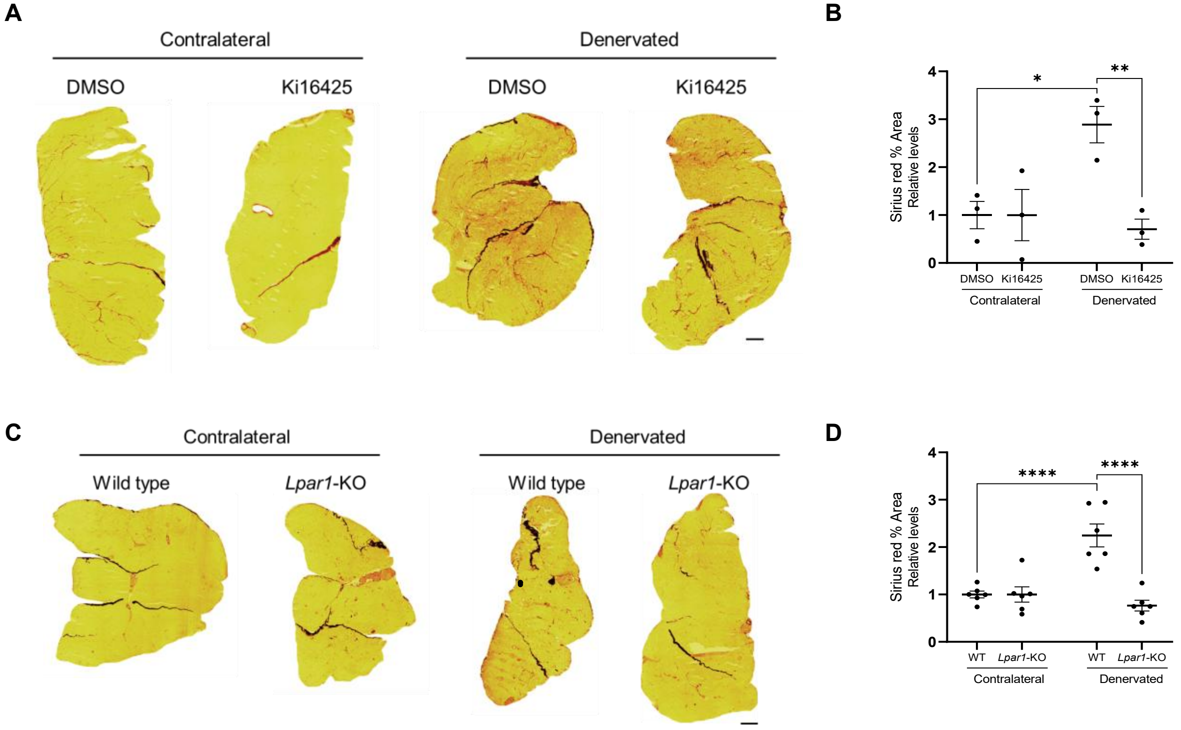

Supplementary Figure 2: Pharmacological inhibition and genetic deletion of Lpar1 reduce denervation-induced collagen accumulation. A: The vehicle (DMSO) (n=3) or Ki16425 (n=3) was administered intraperitoneally to 6-month-old C57Bl/6J mice for three days before denervation and maintained the treatment daily for 2 weeks. Sirius red staining for total collagen. Bar: 400 µm. **B:** Quantification of total collagen positive area (A). **C:** 6-month-old BALB/c (n=6) and *Lpar1*-KO (n=6) mice subjected to unilateral sciatic denervation for 2 weeks. Sirius red staining for total collagen. Bar: 400 µm. **D:** Quantification of total collagen positive area (C). ****P < 0.0001, **P < 0.01, *P < 0.05 with One-way ANOVA test. Values represent means ± SEM.

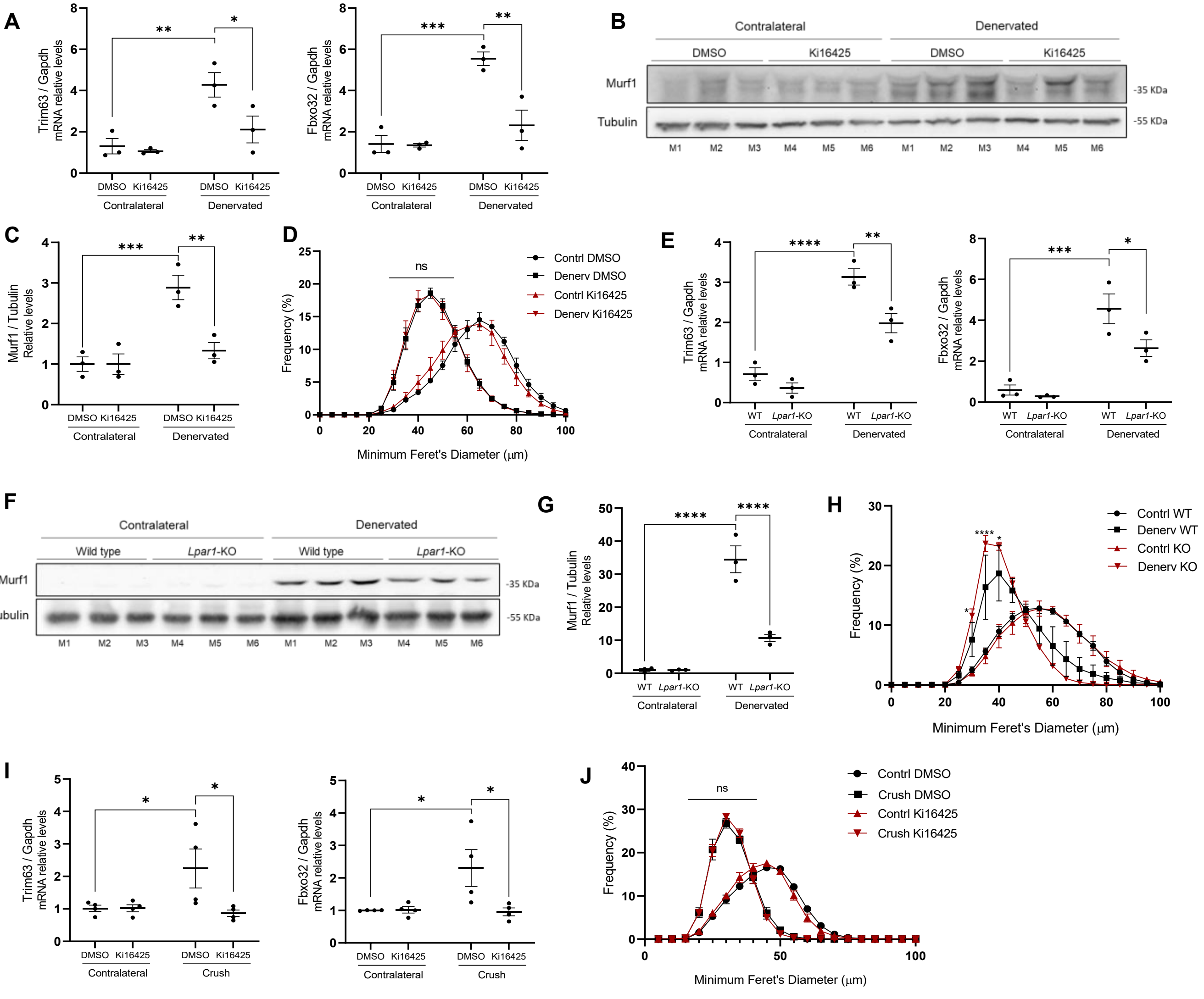

Supplementary Figure 3: LPA signaling is required for the induction of skeletal muscle atrophy markers after denervation. 6-month-old C57Bl/6J mice were treated with vehicle (DMSO) (n=3) or Ki16425 (n=3) for three days before unilateral sciatic denervation. Skeletal muscles from both hindlimbs were collected 2 weeks after denervation. **A:** MuRF1 (*Trim63*) and atrogin-1 (*Fbxo32*) mRNA levels were measured by RT-qPCR. **B:** GST homogenates were subjected to SDS-PAGE and immunoblotted for MuRF1. Tubulin was used as the loading control. **C:** Quantification of B. **D**: Frequency of muscle fiber size (μm). 6-month-old BALB/c (n=3) and *Lpar1*-KO (n=3) mice 2 weeks after being subjected to unilateral sciatic denervation. **E:** Relative mRNA levels of MuRF1 (*Trim63*) and atrogin-1 (*Fbxo32*). **F:** Western blot analysis of MuRF1. Tubulin was used as the loading control. **G:** Quantification of F. **H:** Frequency of muscle fiber size (μm). The vehicle (DMSO) (n=4) or Ki16425 (n=4) was administered intraperitoneally to 3-month-old C57Bl/6J mice for three days before crush surgery, and daily treatment was maintained for 2 weeks. **I:** Relative mRNA levels of MuRF1 (*Trim63*) and atrogin-1 (*Fbxo32*). **J:** Frequency of muscle fiber size (μm). ****$P < 0.0001$, ***$P < 0.001$, **$P < 0.01$, *$P < 0.05$ with One-way ANOVA test. Values represent means ± SEM.

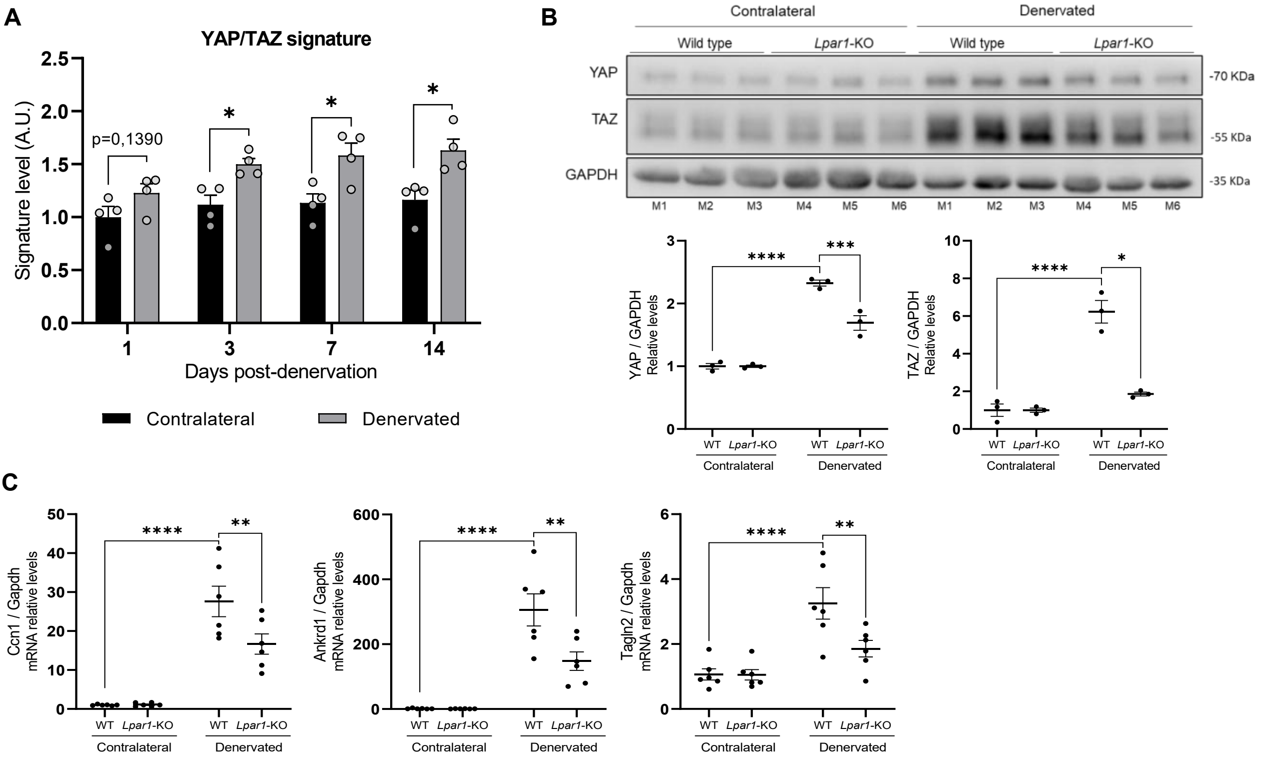

Supplementary Figure 4 *Lpar1* deletion reduces YAP/TAZ accumulation and target gene induction after denervation. A: RNA-seq analysis showing the signature score of YAP/TAZ upregulated genes in denervated and contralateral muscles (1-, 3-, 7-, and 14 days post-surgery) (70, 71). *P < 0.05 with Student´s t-test. Values represent means ± SEM. **B, C:** 6-month-old BALB/c and *Lpar1*-KO mice were subjected to unilateral sciatic denervation for 2 weeks. **B:** Western blot for YAP and TAZ was performed. GAPDH was used as the loading control. Levels of YAP and TAZ were quantified (n=3). **C:** Relative mRNA levels of *Ccn1*, *Tagln2*, and *Ankrd1* (n=6). ****P < 0.0001, ***P < 0.001, **P < 0.01, *P < 0.05, with One-way ANOVA test. Values represent means ± SEM.

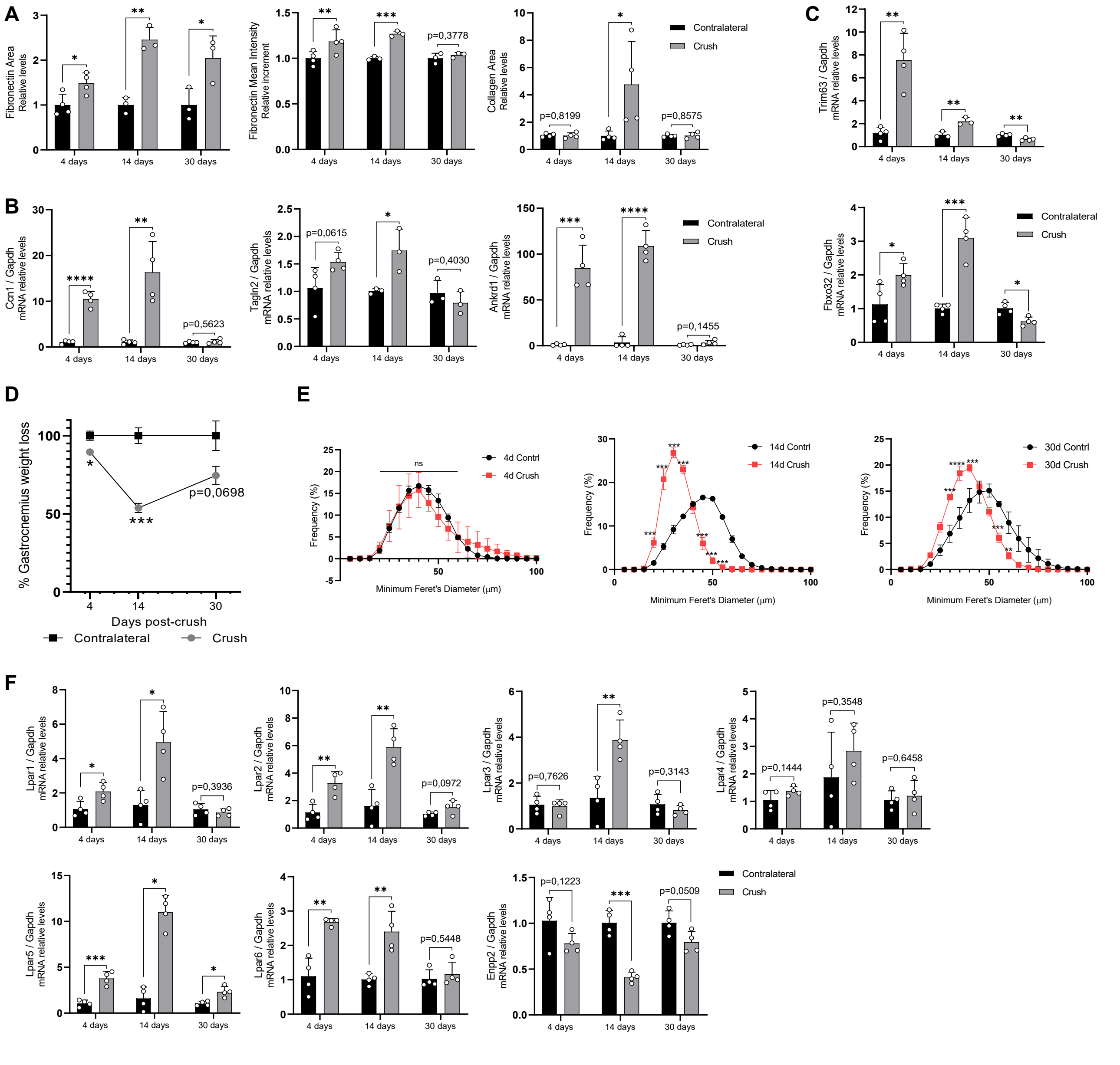

Supplementary Figure 5: Denervation by crush induces a temporally fibrotic response, accompanied by YAP/TAZ, atrophy, and LPA signaling axis gene dysregulation. 3-month-old C57Bl/6J mice were denervated by unilateral crush injury of the sciatic nerve. GST muscles were collected at 4, 14, and 30 days (n=3-4). A: Quantification of fibronectin mean intensity, and fibronectin and total collagen positive area from GTS cross-sections. B: Relative mRNA levels of *Ccn1*, *Tagln2*, and *Ankrd1*. C: Relative mRNA levels of MuRF1 (*Trim63*) and atrogin-1 (*Fbxo32*). D: GST weight of crushed limb expressed as % of weight loss in comparison to de contralateral limb (100%). E: Frequency of muscle fiber size (µm). F: RT-qPCR analysis showing LPARs (*Lpar1* to *Lpar6*), lipid phosphate phosphatases 1, 2, and 3 (*Plpp1*, *Plpp2,* and *Plpp3*), and autotaxin (*Enpp2*) mRNA expression. ****P < 0.0001, ***P < 0.001, **P < 0.01, *P < 0.05 with Student´s t-test (Contralateral vs. Crush). Values represent means ± SEM.
