# Supplementary material for: Lysophosphatidic acid mediates skeletal muscle fibrosis in denervation via activation of YAP/TAZ
Source: JCI Insight. 2026 Apr 22;11(8):e198388. doi: 10.1172/jci.insight.198388 (PMC13135410; doi:10.1172/jci.insight.198388)

Uncropped/Unedited Full Blot Images

Figure 1C

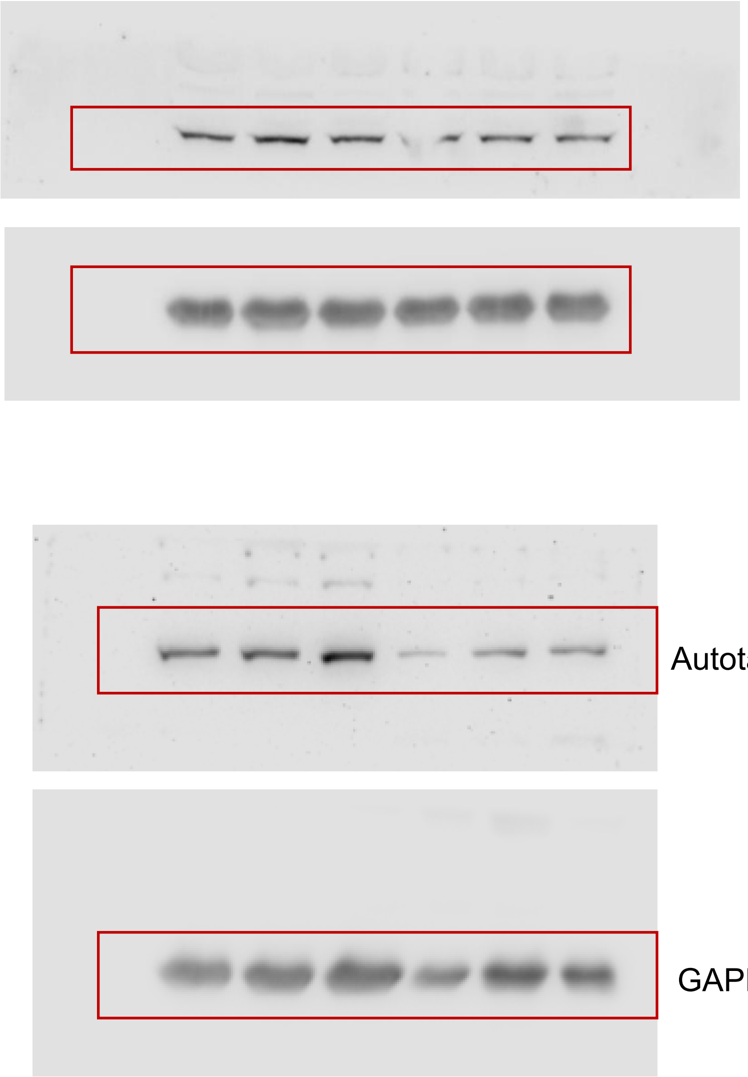

Autotaxin

GAPDH

Autotaxin

GAPDH

Figure 2B

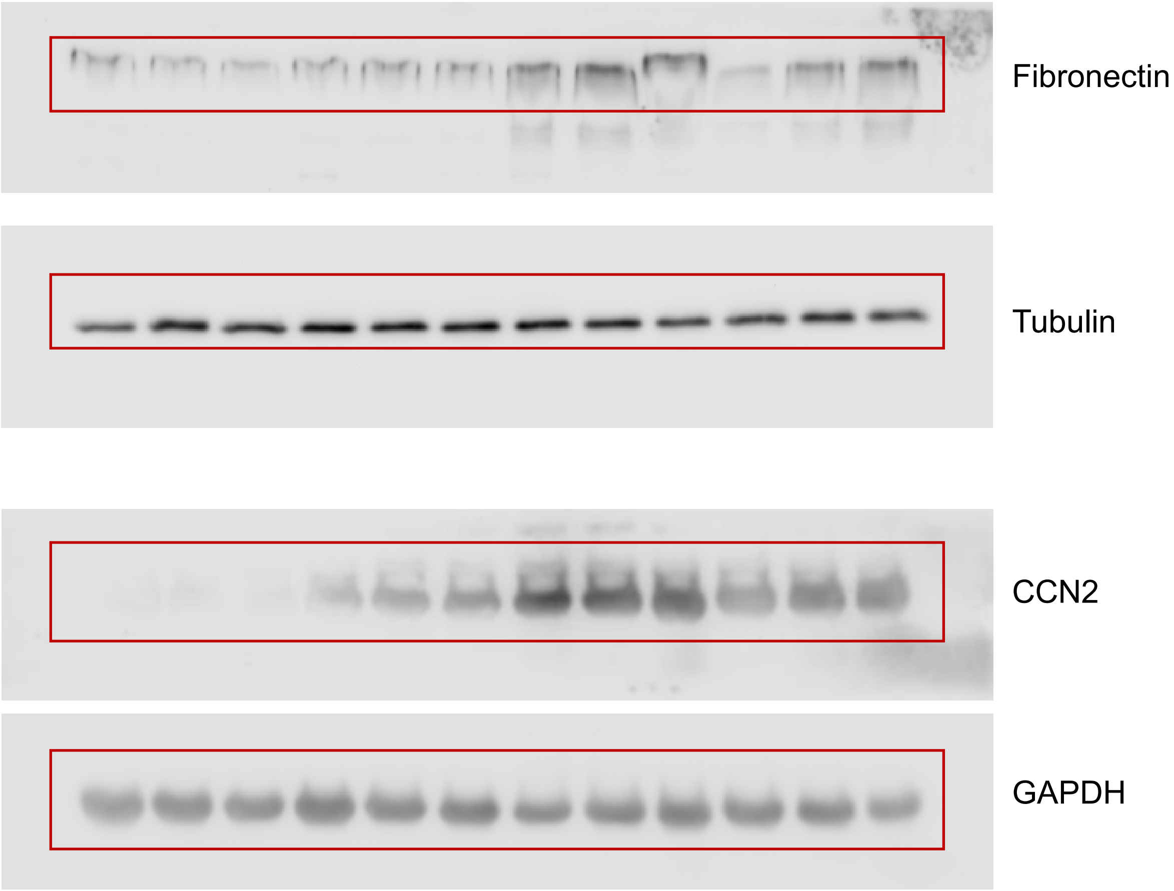

Figure 3B

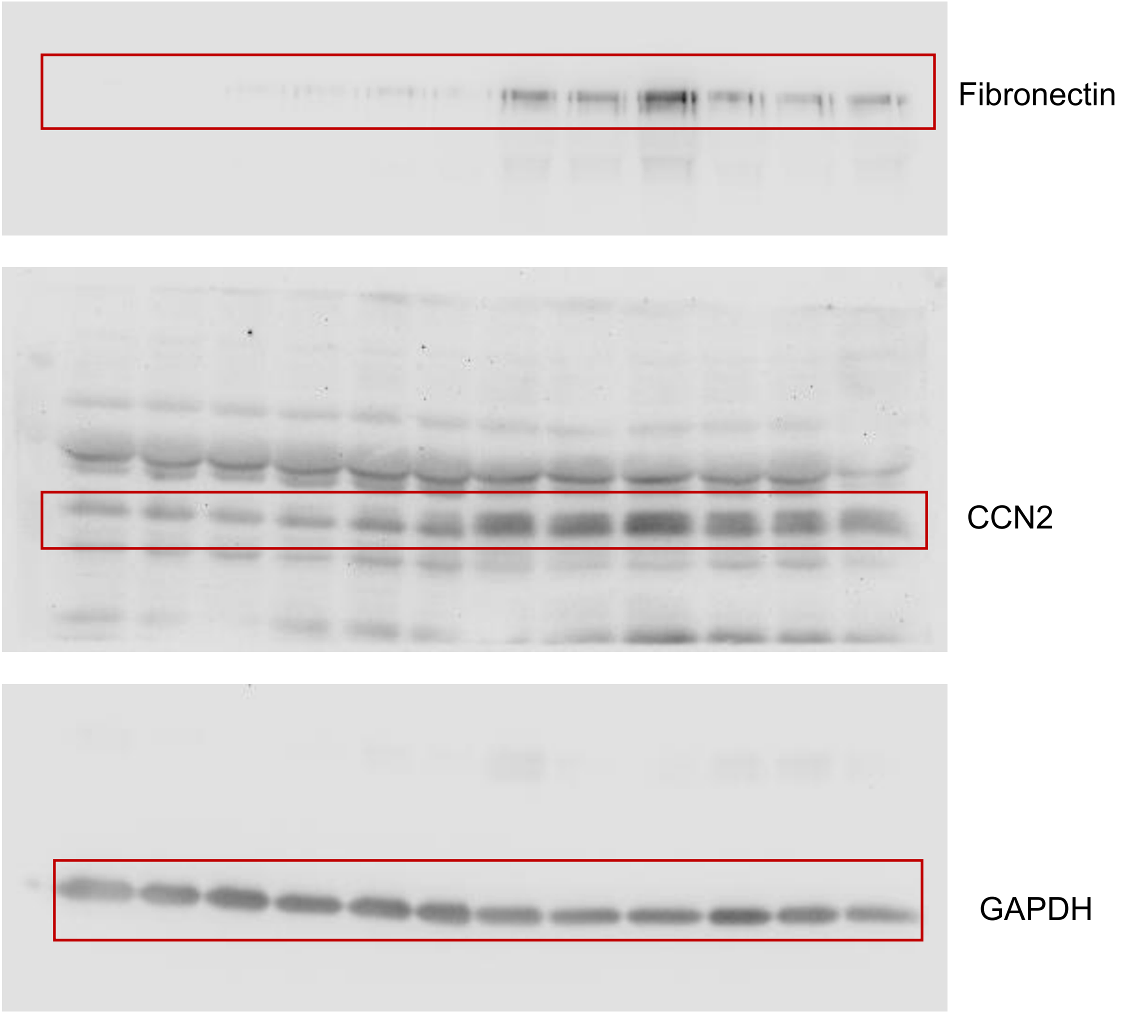

Figure 4A

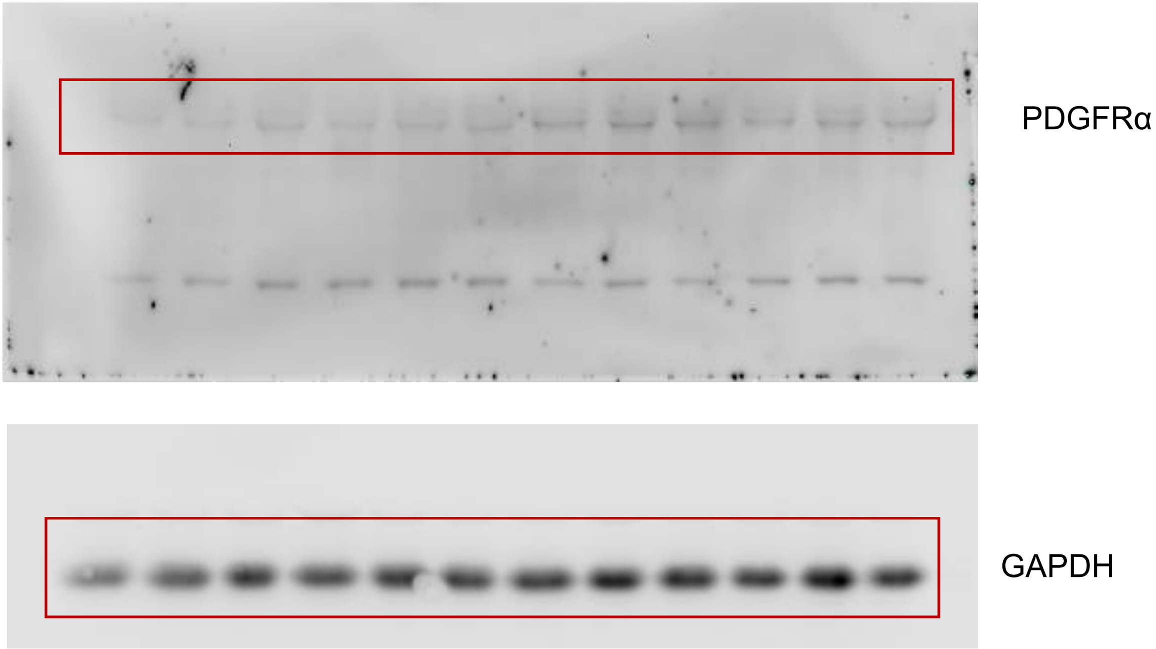

Figure 4B

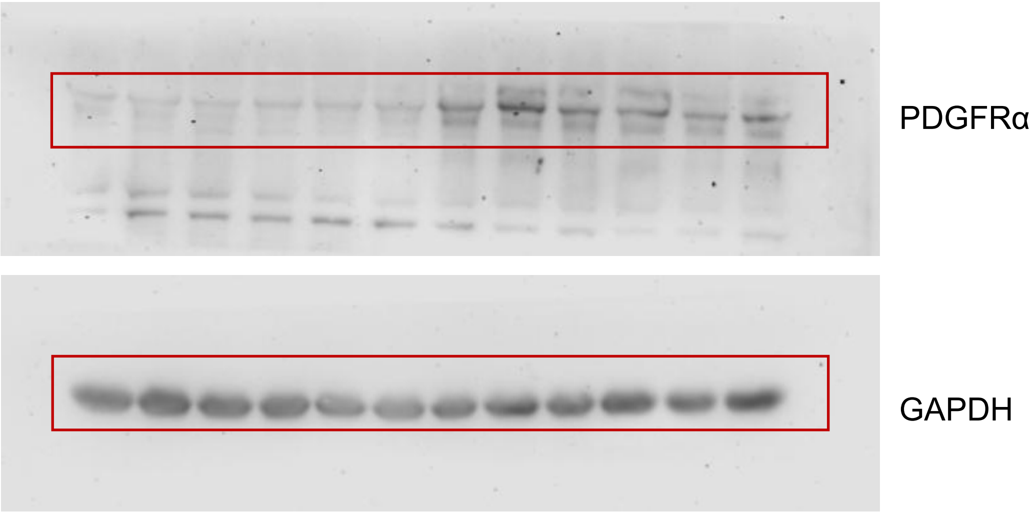

Figure 4C

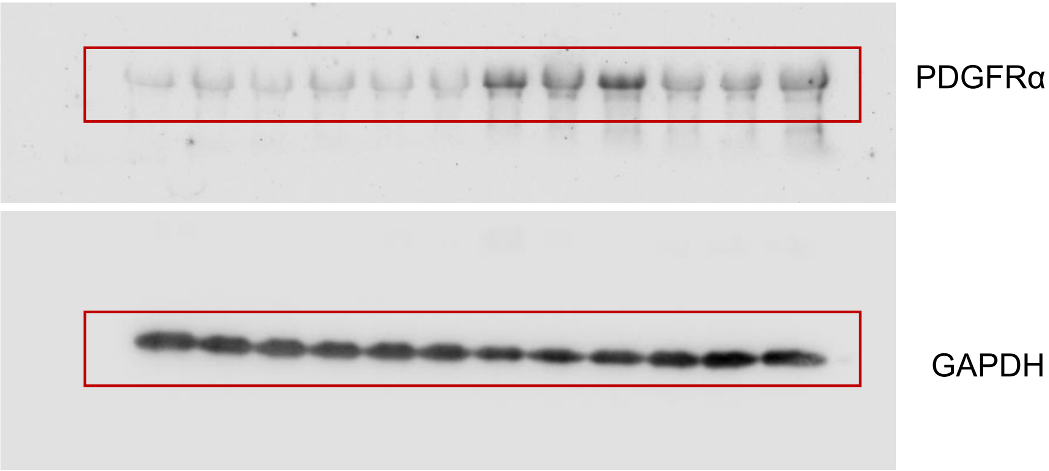

Figure 5A

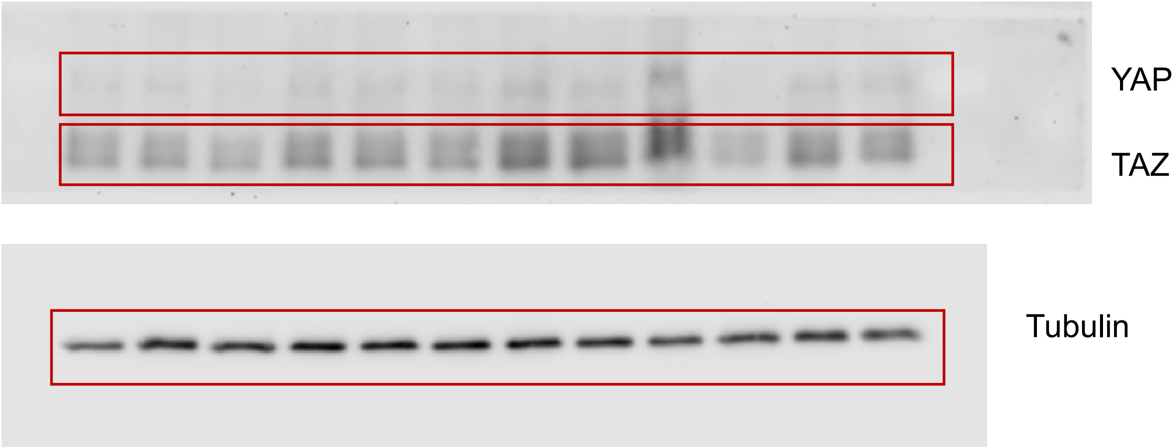

Figure 6B

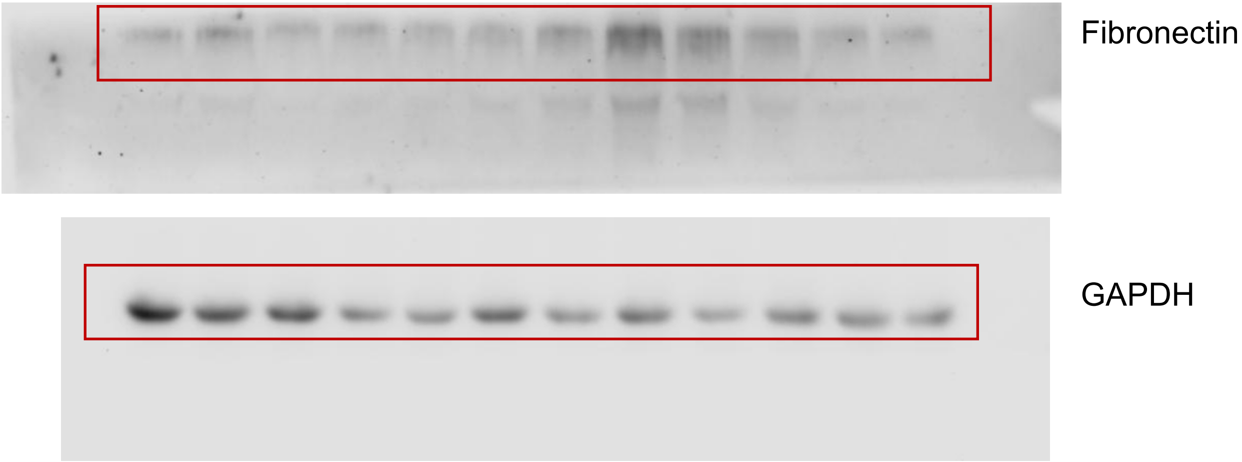

Figure 6D

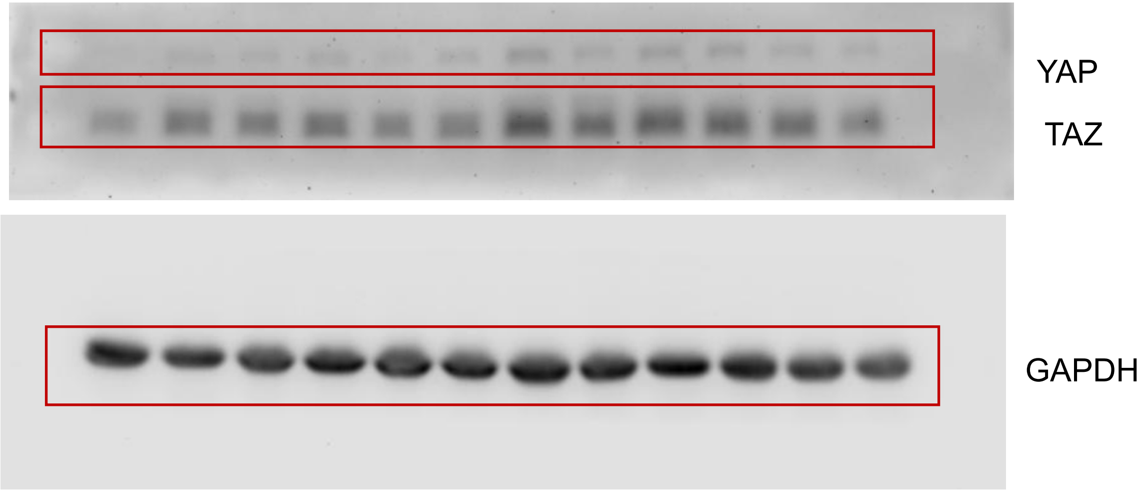

Figure 7C

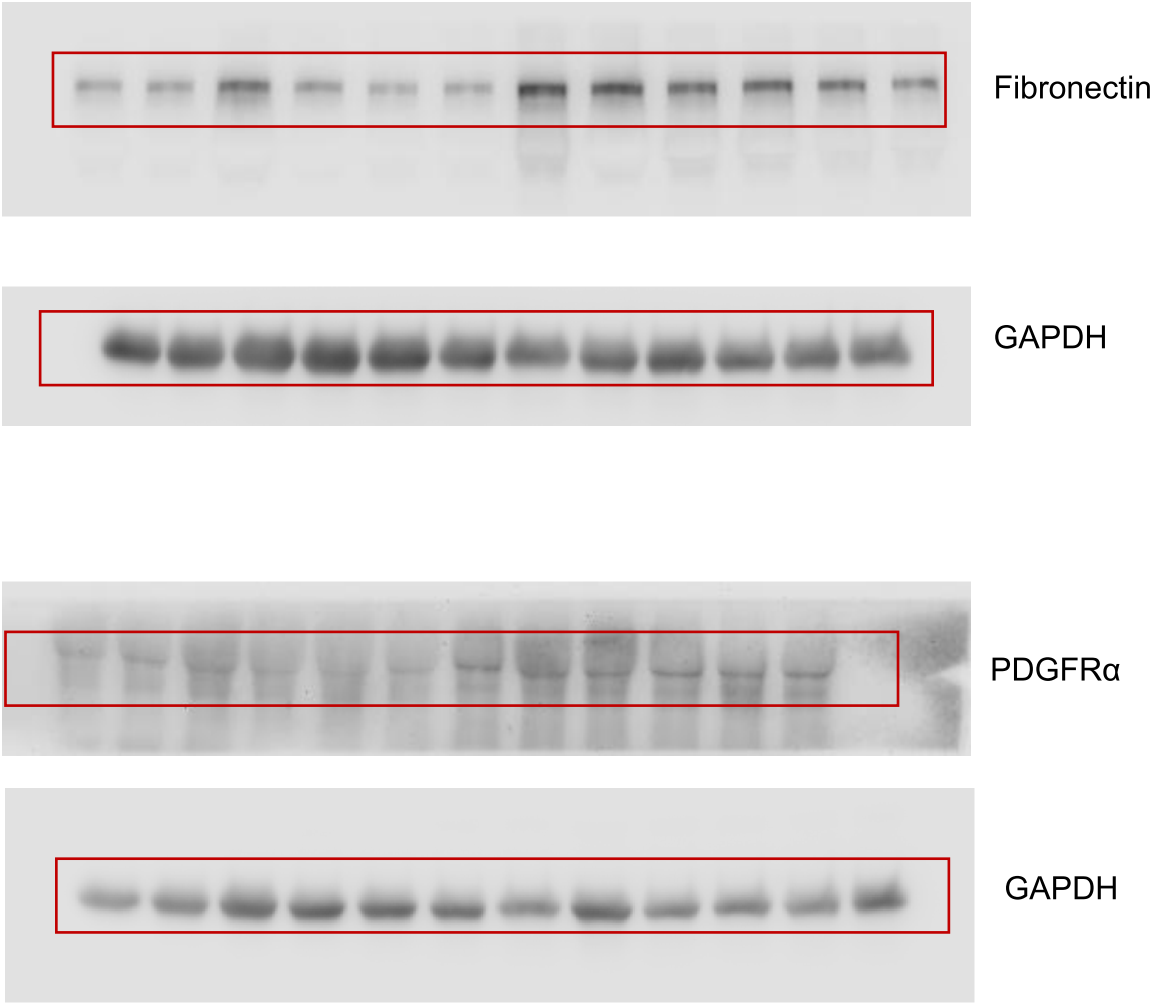

SUPPLEMENTARY

Sup. Figure 3B

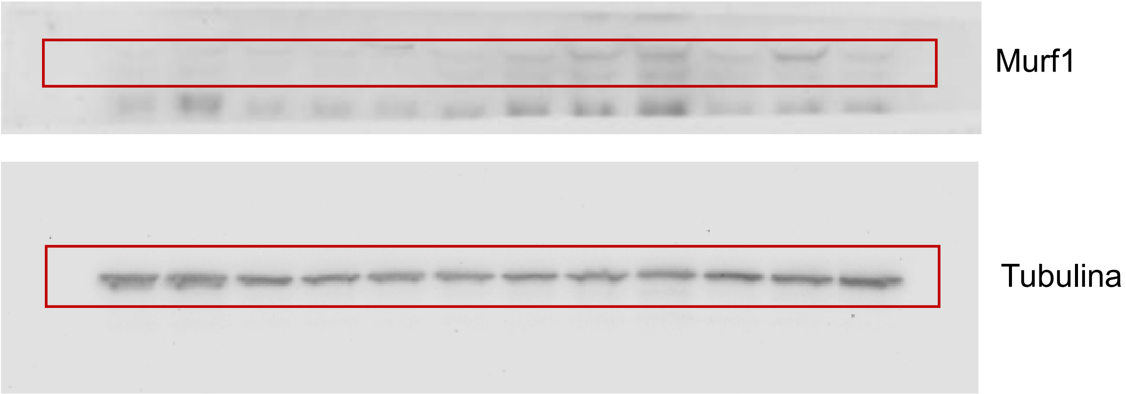

Sup. Figure 3F

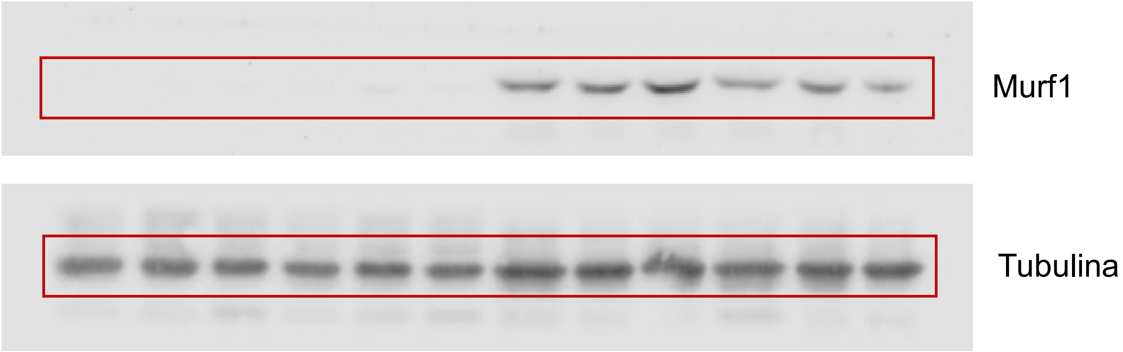

SUPPLEMENTARY

Sup. Figure 4B

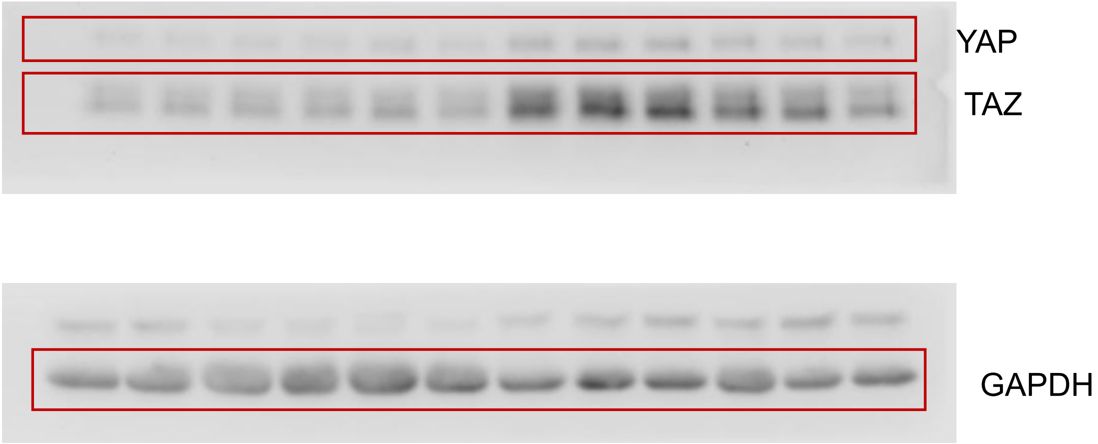

Supplement: Unedited blot and gel images [file jciinsight-11-198388-s118.pdf]
